# Habitat Imaging-Based ^18^F-FDG PET/CT Radiomics for the Preoperative Discrimination of Non-small Cell Lung Cancer and Benign Inflammatory Diseases

**DOI:** 10.3389/fonc.2021.759897

**Published:** 2021-10-06

**Authors:** Ling Chen, Kanfeng Liu, Xin Zhao, Hui Shen, Kui Zhao, Wentao Zhu

**Affiliations:** ^1^ Research Center for Healthcare Data Science, Zhejiang Lab, Hangzhou, China; ^2^ Positron Emission Tomography (PET) Center, The First Affiliated Hospital, School of Medicine, Zhejiang University, Hangzhou, China

**Keywords:** ^18^F-FDG PET/CT, habitat imaging, radiomics, inflammation, non-small cell lung cancer

## Abstract

**Purpose:**

To propose and evaluate habitat imaging-based ^18^F-fluorodeoxyglucose (^18^F-FDG) positron emission tomography/computed tomography (PET/CT) radiomics for preoperatively discriminating non-small cell lung cancer (NSCLC) and benign inflammatory diseases (BIDs).

**Methods:**

Three hundred seventeen ^18^F-FDG PET/CT scans were acquired from patients who underwent aspiration biopsy or surgical resection. All volumes of interest (VOIs) were semiautomatically segmented. Each VOI was separated into variant subregions, namely, habitat imaging, based on our adapted clustering-based habitat generation method. Radiomics features were extracted from these subregions. Three feature selection methods and six classifiers were applied to construct the habitat imaging-based radiomics models for fivefold cross-validation. The radiomics models whose features extracted by conventional habitat-based methods and nonhabitat method were also constructed. For comparison, the performances were evaluated in the validation set in terms of the area under the receiver operating characteristic curve (AUC). Pairwise t-test was applied to test the significant improvement between the adapted habitat-based method and the conventional methods.

**Results:**

A total of 1,858 radiomics features were extracted. After feature selection, habitat imaging-based ^18^F-FDG PET/CT radiomics models were constructed. The AUC of the adapted clustering-based habitat radiomics was 0.7270 ± 0.0147, which showed significantly improved discrimination performance compared to the conventional methods (p <.001). Furthermore, the combination of features extracted by our adaptive habitat imaging-based method and non-habitat method showed the best performance than the other combinations.

**Conclusion:**

Habitat imaging-based ^18^F-FDG PET/CT radiomics shows potential as a biomarker for discriminating NSCLC and BIDs, which indicates that the microenvironmental variations in NSCLC and BID can be captured by PET/CT.

## 1 Introduction

Lung cancer is one of the most fatal and widespread diseases, with a poor 5-year survival rate (1). Non-small cell lung cancer (NSCLC) accounts for 80–85% of lung cancers (2). The histopathological subtypes of NSCLC include large cell carcinoma, adenocarcinoma (ADC), squamous cell carcinoma (SCC), and adenosquamous carcinoma (ASC). Among these subtypes, ASC is a relatively rare NSCLC histopathological subtype whose malignancy contains components of ADC and SCC. The treatment of early-stage NSCLC is normally surgical resection. However, since aspiration biopsy cannot provide 100% sensitivity, several studies have reported that the resection of benign tissue is prevalent because of the aggressive diagnosis and treatment of NSCLC (3–5). On the other hand, aspiration biopsy causes a certain of trauma to patient.

Since malignant tumors mostly present higher glucose metabolism than normal tissue, which is a known hallmark of cancer, they can be detected using ^18^F-fluorodeoxyglucose (^18^F-FDG) positron emission tomography/computed tomography (PET/CT), a metabolic and anatomic imaging system (6, 7). Regarding CT, a tissue can be anatomically analyzed in terms of size, textural heterogeneity, and contour irregulation in CT images (8). From a metabolic perspective, ^18^F-FDG PET can present high FDG metabolism in the region that could be a malignant tumor. Thus, the PET/CT system cannot only provide high sensitivity of radionuclide uptake but also provide precise anatomical information (9). Shim et al. evaluated the preoperative staging accuracy and specificity of ^18^F-FDG PET/CT and found that the performance was significantly better than that of CT alone (10). NSCLC, which presents ground-glass opacity (GGO) in CT, is accompanied by low FDG uptake in PET, thus leading to a false-negative result (11). On the other hand, benign inflammation (e.g., pneumonia, pyogenic abscesses, aspergillosis, and granulomatous diseases), which is also related to increased glucose metabolism, could be a potential false-positive detection in ^18^F-FDG PET (12). False-positive findings are mainly represented by BIDs with high FDG uptake in PET (13, 14). Therefore, a discrimination method is desired for assisting medical physicists and radiologists in identifying whether the high SUV uptake lesion is NSCLC or BID.

Radiomics, which translates medical images into high-throughput quantitative features for analysis, has been applied in a number of clinical studies (15–17). Tumors reveal genomic and phenotypic heterogeneity, which can be reflected in medical images, thus leading to the quantification of the textural and metabolic variations in tumors through PET and CT. Furthermore, PET/CT radiomics, which mines not only textural features but also metabolic features from PET/CT images, has been applied to various potential clinical applications. Lovinfosse et al. studied the prognostic value of baseline ^18^F-FDG PET/CT radiomics, and their results showed a strong predictive ability for survival in patients with locally advanced rectal cancer (18). Antunovic et al. developed and evaluated a PET/CT radiomics model for predicting pathological complete response to neoadjuvant chemotherapy in patients who had locally advanced breast cancer (19). Mu et al. built a PET/CT radiomics signature to predict the outcomes of NSCLC patients treated with checkpoint blockade immunotherapy (20). The above studies employed a conventional radiomics feature extraction method, i.e., radiomics features are extracted based on the whole tumor, which implicitly assumes that the whole tumor shows a consistent heterogeneous pattern. However, intratumoral heterogeneity exists and can be further revealed in medical images (21–23). For example, a whole tumor mass can be divided into well-, moderately, and poorly differentiated volumes, wherein poorly differentiated volumes show significant biological aggressiveness, which makes them different from other well-differentiated tumor volumes, and displays different kinds of heterogeneous pattern. Habitat is a term used to describe these regionally and heterogeneously distinct volumes, while habitat imaging refers to obtaining these volumes (23). Recently, some researchers have started to apply habitat imaging to PET/CT radiomics and have shown competitive performance in their tasks. Wu et al. proposed a robust habitat generation method by clustering and validated it in PET/CT habitat radiomics (24). According to this habitat generation method, Xu et al. built a habitat-based PET/CT radiomics method for predicting progression-free survival in patients with nasopharyngeal carcinoma (25). Wu et al. developed prognostic models by PET/CT habitat radiomics to identify whether more aggressive treatment is needed for patients with locally advanced cervical cancer treated with chemoradiotherapy (26). Different from the abovementioned clustering-based habitat generation method, they used Otsu thresholding (27) to obtain habitats. The thresholding-based habitat generation method fixes the number of habitats for each tumor. However, this thresholding-based method is not suitable, since assuming that the habitat number of NSCLC and BID are totally the same is unreasonable. In addition, the conventional clustering-based habitat generation method fixes the number of supervoxels to be clustered. The supervoxels of a small tumor are aggregated by fewer voxels than those of a large tumor, which suffers from the limited resolution of the image. To improve the issues mentioned above, we refine the clustering-based habitat generation method by adaptively setting the number of supervoxels for each tumor. Furthermore, we use the global signal values instead of the local signal values for normalization. To our knowledge, this is the first study to compare clustering- and thresholding-based habitat generation methods.

Therefore, in this study, we aim to develop and evaluate habitat imaging-based ^18^F-FDG PET/CT radiomics models for discriminating NSCLC and BID, which helps medical physicists and radiologists better diagnose NSCLC by PET/CT images.

## 2 Materials and Methods

**Figure 1:**
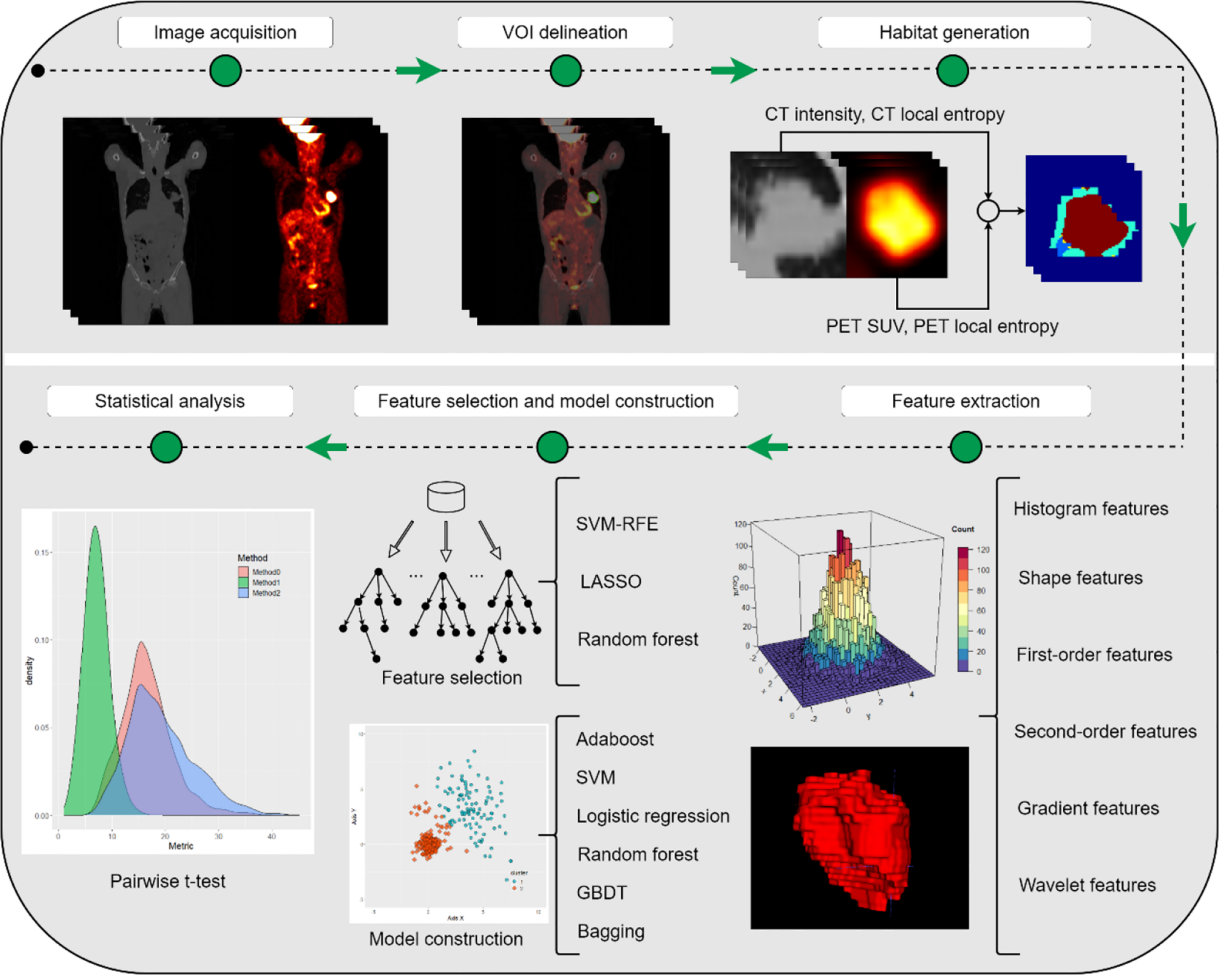
The whole workflow of our study.

This study was approved by the institutional review board, and the requirement for informed consent was waived since the data were analyzed retrospectively and anonymously. The whole workflow of our study is depicted in **Figure 1**.

### 2.1 Image Data

Between January 1, 2015 and February 28, 2021, patients who had scanned PET/CT at the PET Center, the First Affiliated Hospital, School of Medicine, Zhejiang University, underwent aspiration biopsy or surgical resection to obtain histopathological results. The patients were enrolled based on the following inclusion criteria: (i) histopathological results of the lesion were available; (ii) NSCLC was primary and nonmetastatic; and (iii) NSCLC staging was between I and III. A total of 317 lesions were included in our study: 12 had ASC, 126 had ADC, 87 had SCC, and 92 had BID. The demographic information of the enrolled patients is summarized in **Table 1**. All image data were acquired on a Biograph 16 PET/CT system (Siemens Healthineers, Hoffman Estates, IL, USA). The PET images were reconstructed using the iterative algorithm with four iterations and eight subsets. A Gaussian filter with FWHM of 6.0 mm was applied to postprocessing. The convolutional kernel for CT reconstruction was B31f. The CT tube voltage was 120 kV, and the CT tube current was 207.5 ± 57.6 mA. The CT exposure was 105.1 ± 29.2 mAs. All lesions were semiautomatically segmented by two experienced radiologists under mutual consensus using ITK-SNAP 3.6.0 (28). The pixel spacing and slice thickness of each CT image were 1.00 ± 0.08 and 4.93 ± 0.44 mm/voxel, respectively. The pixel spacing and slice thickness of each PET image were 4.06 ± 0.00 and 4.93 ± 0.44 mm/voxel, respectively. Bilinear interpolation was applied to PET and CT images to ensure that they had the same voxel spacing of 1 × 1 × 1 mm^3^.

**Table 1:** Demographic information of the enrolled patients.

		BID	NSCLC		
			ASC	ADC	SCC
**Tumor Number**	92		12	126	87
**Age**	59.2 ± 10.8		60.5 ± 8.4	62.9 ± 9.2	64.9 ± 7.9
**Gender**					
** Female**	37		3	71	3
** Male**	55		9	55	84
**Weight (kg)**	62.4 ± 13.0		65.7 ± 7.9	60.6 ± 9.3	63.9 ± 10.1
**Tumor Diameter (mm)**	61.6 ± 55.9		69.7 ± 80.8	45.6 ± 40.9	61.6 ± 33.0
**Tumor Stage**					
** I**	38		4	62	13
** II**	15		4	32	26
** III**	39		4	32	48

All enrolled PET/CT data were preprocessed before training and testing. Regarding CT, the windowing method was applied to images with customized upper and lower gray level boundaries so that the specific structure could be emphasized. By altering the window width and window level, the contrast and brightness of the image were changed accordingly. Referring to lung cancer diagnosis by CT, radiologists usually use the lung window (window width, 700 HU; window level, −600 HU) to accentuate the lung parenchyma for assessment, including areas of pulmonary vascular structure and consolidation. Furthermore, radiologists normally employ the mediastinal window (window width, 300 HU; window level, 40 HU) to evaluate the mediastinal structures and chest wall, thus leading to the recognition of structures of the mediastinum from enlarged lymph nodes or other masses. Therefore, a customized window (window width, 1,140 HU; window level, −380 HU), which merges the mediastinal window and lung window, was used for preprocessing so that the redundant information could be eliminated and the target region could be enhanced. Regarding PET, all PET images were converted from activity to standardized uptake value (SUV) for the purpose of quantification.

### 2.2 Habitat Generation

#### 2.2.1 Clustering-Based Method

Our clustering-based habitat generation method was adapted from the multiparametric intratumor partitioning method proposed by Wu et al. (24). The main difference between the conventional and adapted method is the hypervolume generation. The conventional method fixed the number of hypervolume for each tumor, while the adapted method fixed the size of hypervolume so each tumors can have adaptive numbers of hypervolume.

The first step is to generate a hypervolume whose dimensionality is feature × depth × height × width. Along the feature axis, we need to determine the size of the feature vector. In our case, we followed the four-dimensional (4D) feature vector, which is comprised of PET SUV, CT intensity, PET local entropy, and CT local entropy (24, 25). In detail, the local entropy for PET and CT was computed within a small 9 × 9 × 9 neighborhood. For example, if the size of the input volume of interest (VOI) is 64 × 64 × 64 for both PET and CT, the size of the resulting hypervolume of the feature should be 4 × 64 × 64 × 64; thus, the first dimension of this hypervolume is a feature vector, which consists of PET SUV, CT intensity, PET local entropy, and CT local entropy. The next step is to individually cluster the voxel for each VOI, and thus, supervoxels (i.e., internal clusters for each VOI) can be obtained. The k-means clustering algorithm was applied to voxel values (PET SUV, CT intensity, PET local entropy, and CT local entropy) under the Euclidean distance measurement. The intensity of the supervoxel is characterized by averaging feature vectors. With respect to the conventional clustering-based method, the cluster numbers of each VOI are set to the same number. The normalization of the 4D feature vector is performed using the maximum and minimum values among the 9 × 9 × 9 neighborhoods. Regarding our adapted clustering-based method, we fix the volume of supervoxels to be 729 mm^3^ and then calculated how many supervoxels need to be clustered. In addition, global minimum and maximum values were used for normalization of the feature vector, which helps measure the distance better. The last step is to cluster supervoxels from all VOIs to form multiple habitats. The k-means clustering algorithm was applied to aggregate all supervoxels to form habitats. The number of habitats was tested from 2 to 10 to determine the optimized number of habitats with the highest evaluation metric, the Calinski–Harabasz index (29).

#### 2.2.2 Thresholding-Based Method

The Otsu algorithm was used for thresholding-based habitat generation. This algorithm determines the threshold by maximizing interclass variance, or equivalently, by minimizing intraclass variance. Otsu thresholding was applied to PET and CT images, and thus, two thresholds of PET and CT were obtained for separating habitats, i.e., PET_high_, PET_low_, CT_high_, and CT_low_. Therefore, a total of four habitats were generated for each tumor: PET_high_ ∩ CT_high_, PET_high_ ∩ CT_low_, PET_low_ ∩ CT_high_, and PET_low_ ∩ CT_low_.

### 2.3 Feature Extraction

Feature extraction was performed on each habitat for each modality. The PyRadiomics Python package version 3.0.1 (30), which is based on the Image Biomarker Standardization Initiative (31, 32), was used to extract 107 basic radiomics features, including first-order, shape, gray level co-occurrence matrix (GLCM), gray level size zone matrix, gray level run length matrix (GLRLM), neighboring gray tone difference matrix (NGTDM), and gray level dependence matrix (GLDM) features (30). Note that the bin widths of CT and PET were set to 25 and 0.25, respectively. Furthermore, co-occurrence of local anisotropic gradient orientations (CoLIAGe) was employed to extract 390 features from a gradient perspective (33). A total of 432 wavelet-local binary pattern (LBP) features, which show competitive performance in capturing cancerous heterogeneity, were the first-order features extracted from images that were orderly processed by discrete wavelet transformation and LBP (34). The details of radiomics features can be found in the Supplementary Information.

### 2.4 Feature Selection and Model Construction

For feature selection, Spearman’s rank correlation coefficient was calculated to eliminate redundant features whose coefficients were >0.99, and then, a hypothesis test was employed to select the features that were significantly associated (p <.05) with the predictive label, i.e., NSCLC and BID. In detail, the Shapiro–Wilk test and Levene’s test were used to test whether the features satisfied a normal distribution and whether the variances in features were homogeneous. If both of these tests satisfied p <.05, a t-test was applied to select the significantly associated features; otherwise, the Mann–Whitney U-test was performed. Afterwards, three different model-based feature selection methods were applied to further select the significant features. Support vector machine recursive feature elimination (SVM-RFE), random forest, and least absolute shrinkage and selection operator (LASSO) regression were used as the model-based feature selection methods. The selected features were used to develop radiomics models, and the classifiers we used included SVM, random forest, gradient boosting decision tree (GBDT), logistic regression, AdaBoost, and bagging. Hence, the resulting number of combinations for constructing radiomics models was 18.

Fivefold cross-validation was performed in model construction, and the evaluation metrics, which included the area under the receiver operating characteristic (ROC) curve (AUC), accuracy, sensitivity, and specificity, were obtained by averaging the values among five folds. Note that the optimized threshold was determined by the highest F1 score in the training set. The following kinds of radiomics models were compared: (i) conventional nonhabitat radiomics; (ii) conventional thresholding-based habitat radiomics; (iii) conventional clustering-based habitat radiomics; (iv) adapted clustering-based habitat radiomics; (v) adapted clustering-based habitat combined with conventional nonhabitat radiomics; (vi) conventional thresholding-based habitat combined with conventional nonhabitat radiomics; and (vii) combination of adapted clustering-based habitat, conventional thresholding-based habitat, and conventional nonhabitat radiomics. In our study, we averaged the predictive value of all habitats in a tumor to obtain the comprehensive predictive value.

### 2.5 Statistical Analysis

There were three feature selection methods and six classifiers and thus leading to 18 radiomics models, which can be constructed for each kind of habitat generation method. In addition, all evaluation metrics were averaged in terms of five folds. To evaluate the performance for different habitat-based radiomics, a pairwise t-test was performed to evaluate whether there was significant improvement between these methods.

## 3 Results

### 3.1 Habitat Characteristics

**Figure 2:**
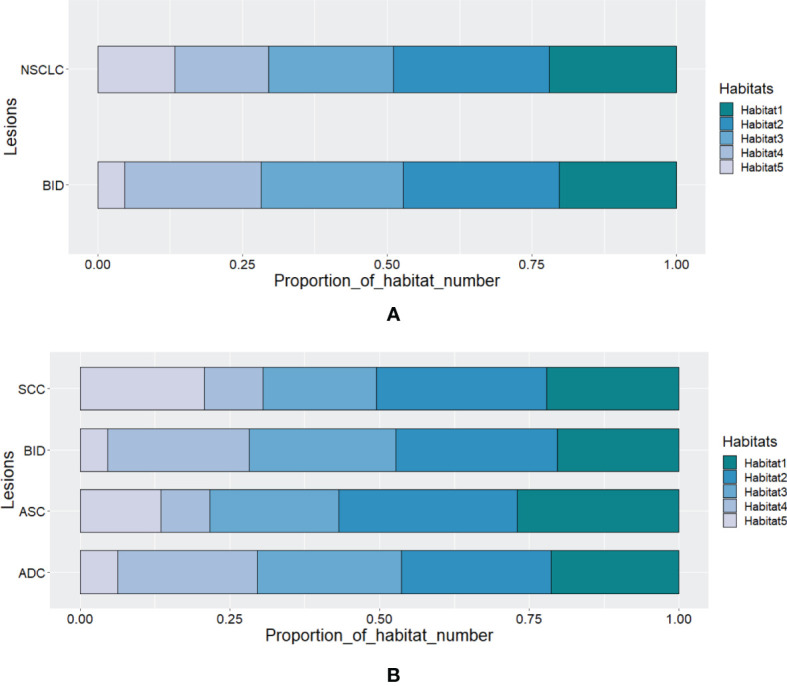
The distribution of habitat number for **(A)** BID and NSCLC and for **(B)** SCC, BID, ASC, and ADC.

**Figure 3:**
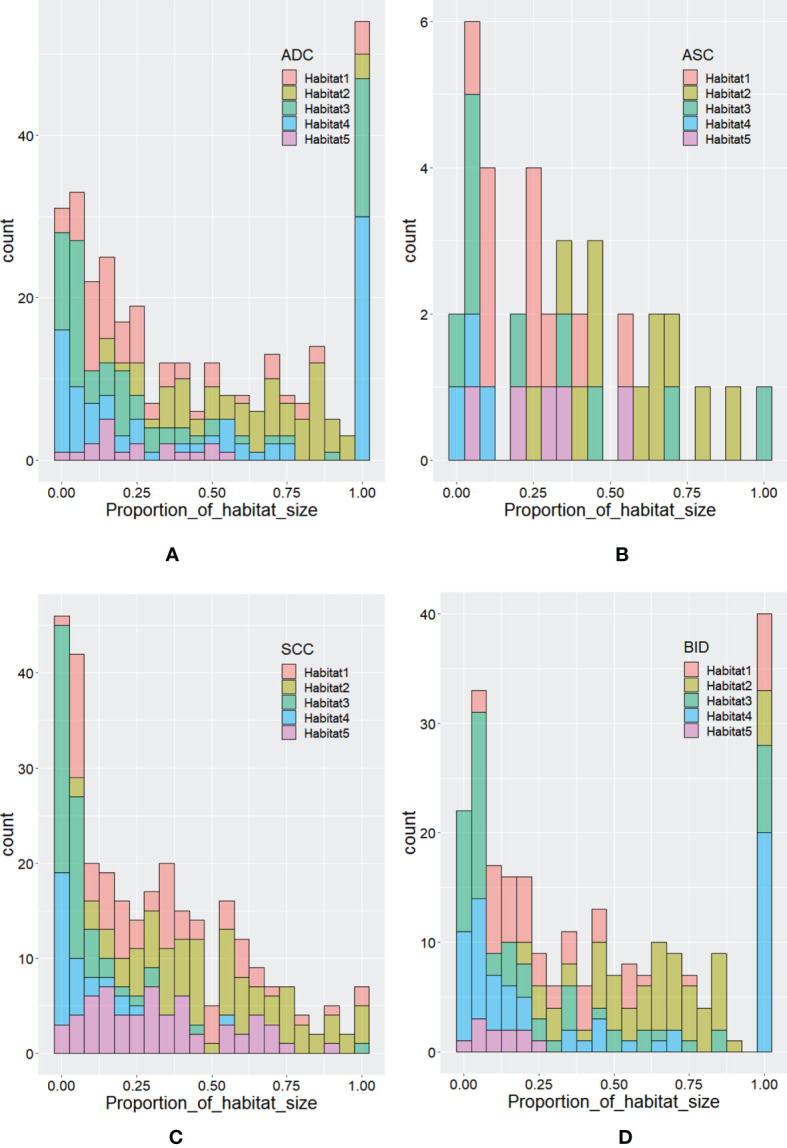
The distribution of habitat size proportion for **(A)** ADC, **(B)** ASC, **(C)** SCC, and **(D)** BID.

According to Calinski–Harabasz index, the optimized numbers of habitats were 5 and 2 for adapted and conventional clustering-based habitat generations in our study, respectively. Regarding our adapted clustering-based method, we adaptively set the number of habitats for each tumor. The distribution of habitat number for each type of lesion is summarized in **Figure 2**. From **Figure 2A**, we can see that the habitat number proportions of habitats 1–3 between NSCLC and BID are similar, while habitats 4 and 5 show differences between NSCLC and BID. Furthermore, NSCLC was split into histopathological subtypes, including ADC, ASC, and SCC, as shown in **Figure 2B**. Specifically, **Figure 2B** shows that the habitat number distribution of BID is very close to that of ADC. **Figure 3** shows the stacked histograms of habitat size for each NSCLC histopathological subtype and BID. The vertical and horizontal axes represent the occurrence of a specific habitat and the proportion of the habitat size and the whole tumor size, respectively. The size proportion represents the ratio of the size of habitat to the size of whole VOI. From **Figure 3**, we can see that (a) ADC could appear as a single habitat as habitats 1–3, while the occurrence of habitat 5, which merely appears as a small region, is very low; (b) ASC mainly appears as habitats 2 and 3 in terms of the size proportion and unlikely appears as habitat 4; (c) SCC tends to appear as multiple habitats, with the highest frequency of habitat 5 compared to the others; and (d) the distribution of BID is very similar to that of ADC, which shows a similar phenomenon as that in **Figure 3B**.

### 3.2 Performance Evaluation of Habitat Imaging-Based Radiomics

**Figure 4:**
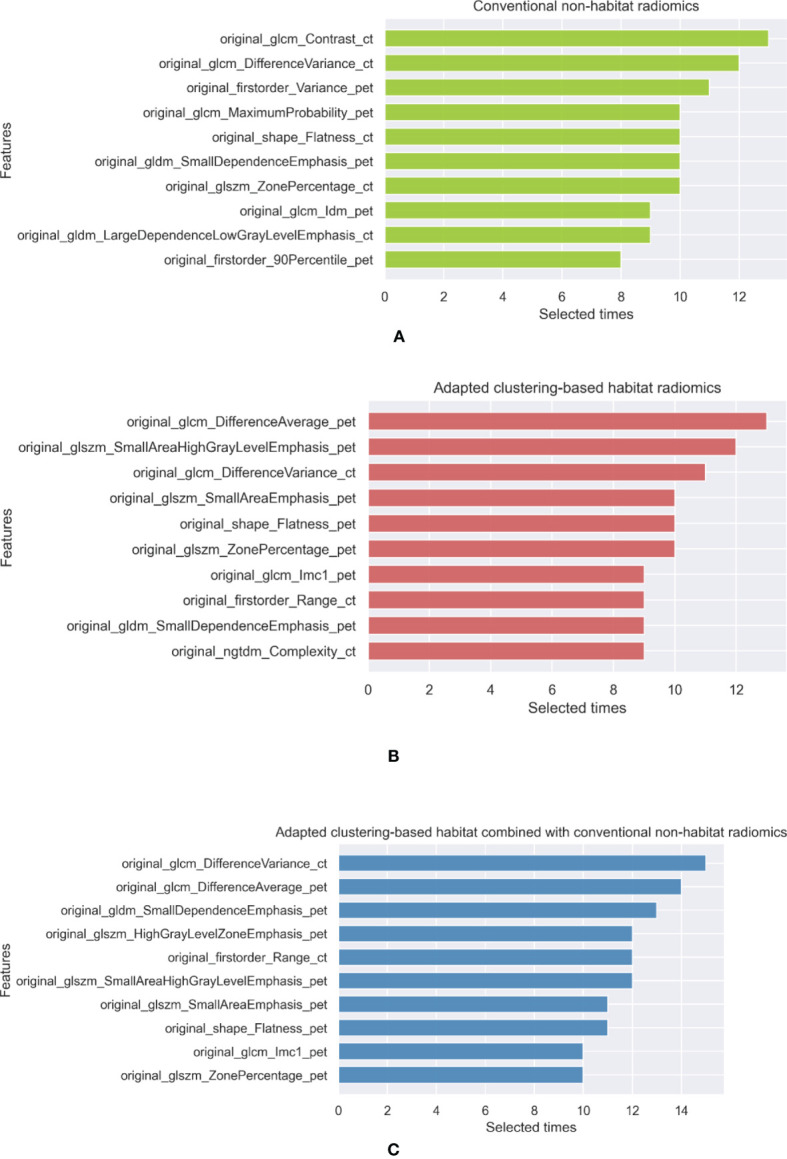
The top 10 selected features of **(A)** conventional nonhabitat radiomics, **(B)** adapted clustering-based habitat radiomics, and **(C)** their combination.

Radiomics features were extracted from these five habitats. Since stratified sampling was applied to fivefold cross-validation, the training, validation, and testing sets had the same proportions of ADCs, ASCs, SCCs, and BIDs, which suggests that these subsets of data have similar lesion distributions. Since we used three feature selection methods for fivefold cross-validation, feature selection was applied 15 times to develop habitat radiomics. Thus, the maximum number of selected times for a feature is 15. We summarized the top 10 selected features for each habitat radiomics. The selected features of conventional nonhabitat radiomics, adapted clustering-based habitat radiomics, and their combination are shown in **Figure 4**. The top 10 selected features for the other habitat methods are summarized in **Supplementary Figure 1** of the Supplementary Information. Regarding conventional nonhabitat radiomics, the top 10 selected features included 4 features from CT and 5 features from PET. There were three histogram features, one shape feature, and six second-order features. Regarding adapted clustering-based habitat radiomics, three and six features were based on CT and PET, respectively. There was one shape feature, one first-order feature, and eight second-order features. Regarding adapted clustering-based habitat radiomics combined with conventional nonhabitat radiomics, only two features were from CT and seven features were from PET. There was one histogram feature, one shape feature, and eight second-order features. Comparing the features extracted from conventional nonhabitat radiomics with those extracted from adapted clustering-based habitat radiomics, we can see that more PET features were extracted, and the top 2 features were changed from CT to PET, which implicitly reveals that the adapted clustering-based habitat can discover more diagnostic potential in PET with the help of CT.

**Figure 5:**
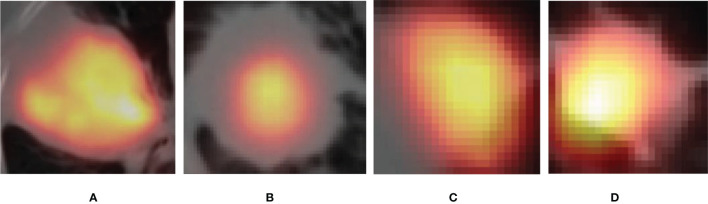
The selected four samples of **(A)** ADC, **(B)** SCC, **(C)** ASC, and **(D)** BID in PET/CT images.

**Figure 6:**
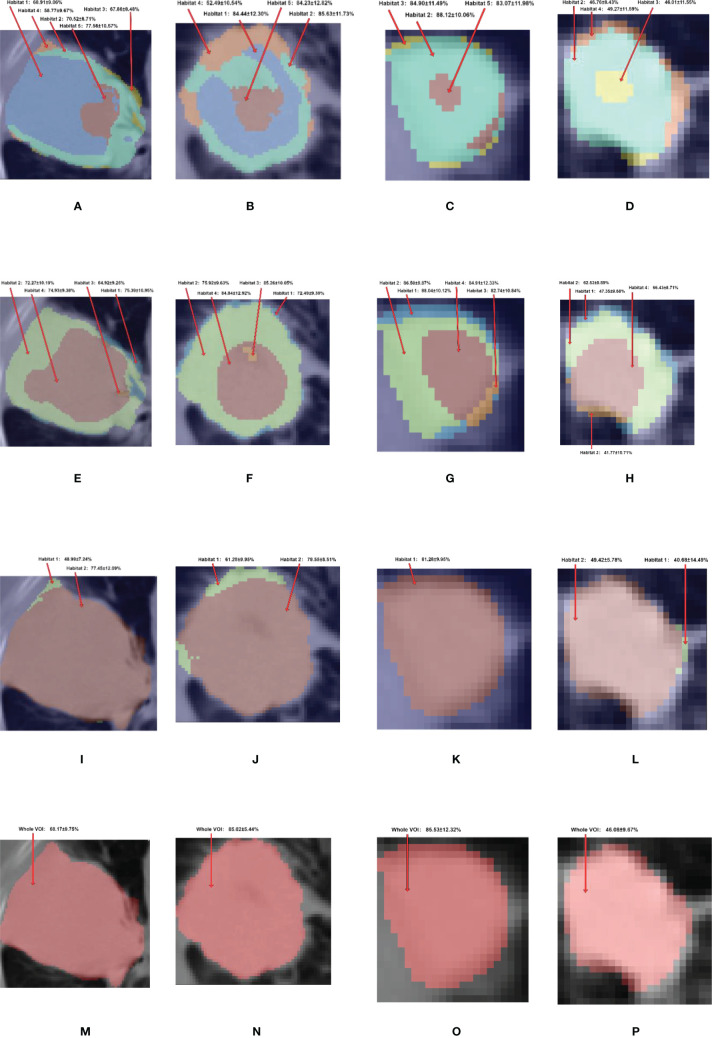
The habitat generated by **(A–D)** adapted clustering-based, **(E–H)** conventional thresholding-based, **(I–L)** conventional clustering-based, and **(M–P)** nonhabitat methods and their corresponding predicted probability. The first, second, third and fourth columns represent ADC, SCC, ASC and BID, respectively. For the adapted clustering-based method, the habitat 1 to 5 are showed in the colors of blue, aquamarine, yellow, orange and red respectively. For the conventional thresholding-based method, habitats 1–4 are showed in colors of blue, green, orange and red, respectively. For the conventional clustering-based method, the habitats 1 and 2 are showed in colors of green and red, respectively.

A total of 6 classifiers and 3 feature selection methods, which form 18 different radiomics pipelines, were used to develop radiomics models. The radiomics features extracted from different habitat generation methods were fed into these pipelines, and then the mean and standard deviation of the evaluation metrics were calculated to evaluate the overall predictive performance. A total of seven habitat imaging-based radiomics methods were compared, as mentioned in Section 2.4. Their diagnostic performance metrics are summarized in **Table 2**. In this table, T1 depicts the pairwise t-test between the adapted clustering-based combined with nonhabitat method and the rest of methods, and T2 illustrates the pairwise t-test between the adapted clustering-based method and the other habitat generation methods. The performance details for the specific classifiers are summarized in **Supplementary Table 1** of the Supplementary Information. The metrics of accuracy, sensitivity, and specificity are based on the optimized threshold obtained from the F1 score. Since we had a highly imbalanced dataset and the three metrics are easily affected by the threshold, the AUC metric, which can comprehensively reflect the performance of binary classification models, was used for the performance comparisons (35). According to **Table 2**, we can see that the features extracted from the adapted clustering-based method combined with the non-habitat method can be used to develop radiomics models that outperform the others, except for the adapted clustering-based radiomics method, in terms of the AUC (p <.05), with a mean AUC of 0.7329. For further analyze the habitat characteristics; four samples were selected in **Figure 5**, and their corresponding habitats with predicted probability are plotted in **Figure 6**.

**Table 2:** Summary of the diagnostic performances for each method.

**Habitat method**	**Training set**				**Testing set**				**P-value**		
	AUC	Acc.	Sens.	Spec.	AUC	Acc.	Sens.	Spec.	T1	T2
**Non-habitat**	0.8751 ± 0.0768	0.8519 ± 0.0752	0.9601 ± 0.0199	0.5867 ± 0.2234	0.6938 ± 0.0190	0.7087 ± 0.0232	0.8486 ± 0.0595	0.3674 ± 0.0836	<.001^*^	<.001^*^
**Conventional thresholding-based**	0.8350 ± 0.0817	0.8460 ± 0.0685	0.9625 ± 0.0161	0.5607 ± 0.2266	0.7090 ± 0.0165	0.7259 ± 0.0194	0.8889 ± 0.0468	0.3256 ± 0.0692	<.001^*^	<.001^*^
**Conventional clustering-based**	0.8259 ± 0.0877	0.8413 ± 0.0702	0.9580 ± 0.0148	0.5381 ± 0.2215	0.6567 ± 0.0223	0.7204 ± 0.0170	0.8826 ± 0.0474	0.3214 ± 0.0826	<.001^*^	<.001^*^
**Adapted clustering-based**	0.8554 ± 0.0749	0.8434 ± 0.0717	0.9562 ± ± 0.0181	0.5706 ± 0.2156	0.7270 ± 0.0147	0.7268 ± 0.0220	0.8330 ± 0.0354	0.3475 ± 0.0931	.084	**–**
**Conventional thresholding-based + Non-habitat**	0.8423 ± 0.0761	0.8552 ± 0.0679	0.9647 ± 0.0141	0.5860 ± 0.2174	0.7124 ± 0.0290	0.7281 ± 0.0124	0.8773 ± 0.0372	0.3590 ± 0.0896	.002^*^	
**Adapted clustering-based + Non-habitat**	0.8653 ± 0.0720	0.8599 ± 0.0698	0.9628 ± 0.0192	0.6091 ± 0.2050	0.7329 ± 0.0170	0.7422 ± 0.0138	0.8974 ± 0.0316	0.3626 ± 0.0743	**-**	
**Adapted clustering-based + Non-habitat + Conventional thresholding-based**	0.8408 ± 0.0628	0.8578 ± 0.0556	0.9586 ± 0.0184	0.5965 ± 0.1727	0.7144 ± 0.0164	0.7306 ± 0.0195	0.8807 ± 0.0295	0.3603 ± 0.0951	<.001^*^	

^*^Significant result.

## 4 Discussion

In this study, three kinds of habitat generation methods were compared. PET and CT radiomics features were extracted from these habitats. Radiomics models were then developed to compare the predictive performance metrics of these habitat generation methods. Since we had a highly unbalanced dataset, with 225 NSCLC cases and 92 BID cases, the evaluation metrics of accuracy, sensitivity, and specificity were highly affected by the discrimination threshold. Thus, we only compared the AUC to evaluate the difference in performance. The adapted clustering-based habitat radiomics model showed a significantly better AUC of 0.7329 ± 0.0170 in the fivefold testing sets than the non-habitat radiomics (p <.001), conventional thresholding-based radiomics (p <.001), and conventional clustering-based radiomics (p <.001) models. This finding implies that adapted clustering-based habitat radiomics has the potential to provide significant values to medical physicists and radiologists and help customize the therapeutic strategy for patients (36), e.g., with the help of habitats, medical physicists and radiologists can focus on the most malignant region for radiotherapy. Furthermore, we can see that habitat radiomics developed by combining adapted clustering-based features with conventional nonhabitat features shows the best predictive performance in terms of evaluation metrics compared with the other combinations.

To further analyze habitat radiomics, we selected four samples of ADC, SCC, ASC, and BID, and their corresponding PET/CT fusion images are shown in **Figure 5**. For these four samples, we plotted the habitats for each of the habitat generation methods, as shown in **Figure 6**. Moreover, the specific predictive values of the models, which were developed by the adapted clustering-based habitat, conventional thresholding-based habitat, and conventional nonhabitat radiomics, are marked accordingly in **Figure 6**. By looking at the habitats of the four samples generated by the adapted clustering-based method (the first row of **Figure 6**), we can see that habitat 2 tends to appear as a shell shape, habitat 4 tends to appear in relatively benign tissue, and habitat 5 tends to appear at the center of the lesion. Habitat 5 only appeared in these three NSCLC samples, while it did not appear in the BID sample. This finding was also supported by **Figure 2D**, which demonstrates that BID had almost no habitat 5, and if there was any sign of habitat 5, the volume was very small. Habitat 1 only appeared in ADC and SCC among the four samples. The four samples in the second row of **Figure 6** were generated based on conventional thresholding-based method. Note that PET_low_ ∩ CT_low_, PET_low_ ∩ CT_high_, PET_high_ ∩ CT_low_, and PET_high_ ∩ CT_high_ represent habitats 1–4, respectively. For these four cases, habitat 4 tends to appear in the tumor core region, while habitats 1 and 2 appeared as outer and inner shell shapes, respectively. Habitat 3 was shown in the tumor core as a small volume. However, all four samples showed a similar habitat pattern because of the characteristics of thresholding-based method. By looking at the third row of **Figure 6**, habitats generated by the conventional clustering-based method only obtained two kinds of habitats. Habitat 1 is very small and only focus on the rim of tumor, while habitat 2 almost occupies the whole volume. Note that we provided an overall predictive value by averaging the predictive values of all habitats for a lesion. Since the adapted clustering-based method is significantly better than the other habitat generation methods, it implicitly shows that the proposed habitat generation method can significantly improve the performance of the radiomics model.

The employment of habitat imaging is due to the subregional heterogeneity and complexity of the tumor microenvironment because there are complex metabolic contacts that appear in cancerous tissues. Given the domain knowledge, cancer cells often have increased glucose metabolism (37). To achieve this, glucose transporters are overexpressed in cancer cells to ensure glucose transportation for oncogenic transformation and progression. Regarding SCC, the histopathological subtype can be indicated by high expression of glucose transporter 1 (GLUT1) (38, 39), e.g., premalignant lesions of bronchial epithelium (40), which can be reflected by a high SUV in PET images. For ADC, the mass can consist of different levels of cell differentiation to demonstrate heterogeneity. Moreover, the expression of glucose transporters in ADC is also heterogeneous, e.g., the poorly differentiated and well-differentiated regions express GLUT1 and sodium-glucose cotransporter-2, respectively (41). On the other hand, inflammation is mainly caused by cells from innate immune system, while cancer cells have a mechanism of cancer immune escape (42). This mechanism allows cancer cells to competitively deprive glucose, an important nutrient, from immune cells (43). Thus, mining the microenvironmental heterogeneity captured by PET/CT can help medical physicists and radiologists better discriminate NSCLCs, and our result demonstrates that habitat imaging is a potential method for mining the heterogeneity of tumors and precise resection. **Supplementary Figure 2** shows the density of habitat size proportion for each kind of lesion, and the differences between the distributions of four kinds of lesions are obvious by visual assessment.

Nevertheless, due to the limitation of sample size and imbalanced class, we used a different method to develop radiomics models. Although different kinds of habitats may have some kinds of difference, we still used habitat as a sample unit to develop models. Nevertheless, the optimal way is to develop tailored radiomics models for each habitat, which means that we should have had five different radiomics models for predicting corresponding habitats. Moreover, to further exploit the potential of habitat imaging, a prospective study should be conducted by jointly utilizing the medical images and the corresponding aligned whole-mount histology images (44). With the help of pathologists, the regions of interest for each NSCLC histopathological subtype can be delineated on histology images. In this case, the habitat generation method is no longer based on the predetermined handcrafted feature vector but is based on the experience of the pathologist. In addition, a thoughtful well-designed model development method can be used to jointly utilize the information from both histology images and PET/CT images and then to cluster the habitats of PET/CT images. Thus, a histopathological subtype can be determined by investigating whether a specific kind of habitat appeared on PET/CT images.

## 5 Conclusion

Habitat imaging is meaningful for dividing a lesion into multiple habitats based on metabolic and anatomic information and further analysis of the lesion. Habitat imaging-based ^18^F-FDG PET/CT radiomics shows potential as a biomarker for discriminating NSCLC and BIDs, which indicates that the microenvironment variations in NSCLC and BIDs can be captured by PET/CT.

## Data Availability Statement

The original contributions presented in the study are included in the article/**Supplementary Material**. Further inquiries can be directed to the corresponding authors.

## Ethics Statement

This study was approved by the institutional review board of Zhejiang University, and the requirement for informed consent was waived since the data were analyzed retrospectively and anonymously.

## Author Contributions

Conceptualization, methodology and investigation: LC, KF, and HS. Writing, review, and editing, LC, KF, XZ, KZ, and WZ. All authors contributed to the article and approved the submitted version.

## Funding

This work was supported by the Department of Education of Zhejiang Province (Y202043388) and the Key Research and Development Program of Zhejiang Province (2021C03029).

## Conflict of Interest

LC, HS, and WZ are employed by Zhejiang Lab.

The remaining authors declare that the research was conducted in the absence of any commercial or financial relationships that could be construed as a potential conflict of interest.

## Publisher’s Note

All claims expressed in this article are solely those of the authors and do not necessarily represent those of their affiliated organizations, or those of the publisher, the editors and the reviewers. Any product that may be evaluated in this article, or claim that may be made by its manufacturer, is not guaranteed or endorsed by the publisher.
