# Supplementary material for: Habitat Imaging-Based 18F-FDG PET/CT Radiomics for the Preoperative Discrimination of Non-small Cell Lung Cancer and Benign Inflammatory Diseases
Source: Front Oncol. 2021 Oct 6;11:759897. doi: 10.3389/fonc.2021.759897 (PMC8526895; doi:10.3389/fonc.2021.759897)
Supplement: Supplementary file 1 [file DataSheet_1.docx]

Supplementary Material

# Supplementary Figures and Tables

## Supplementary Figures


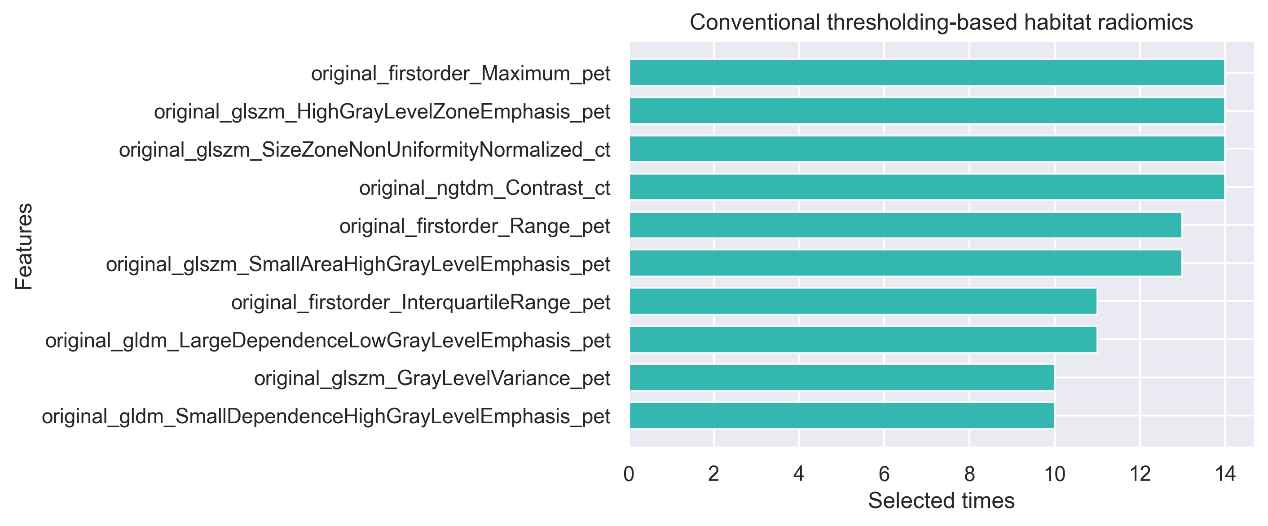


(a)


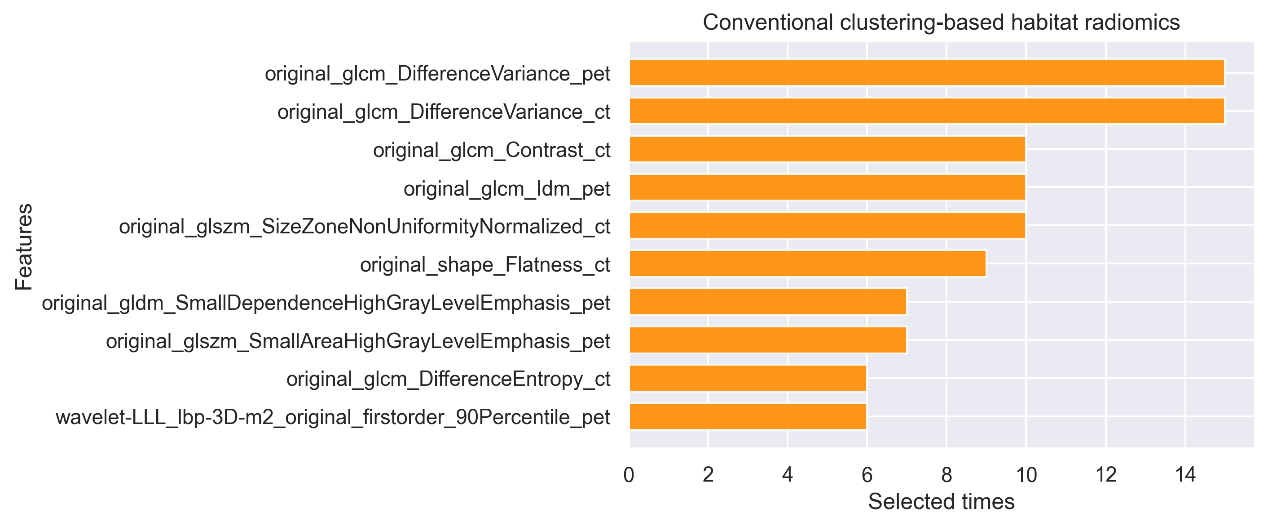
(b)


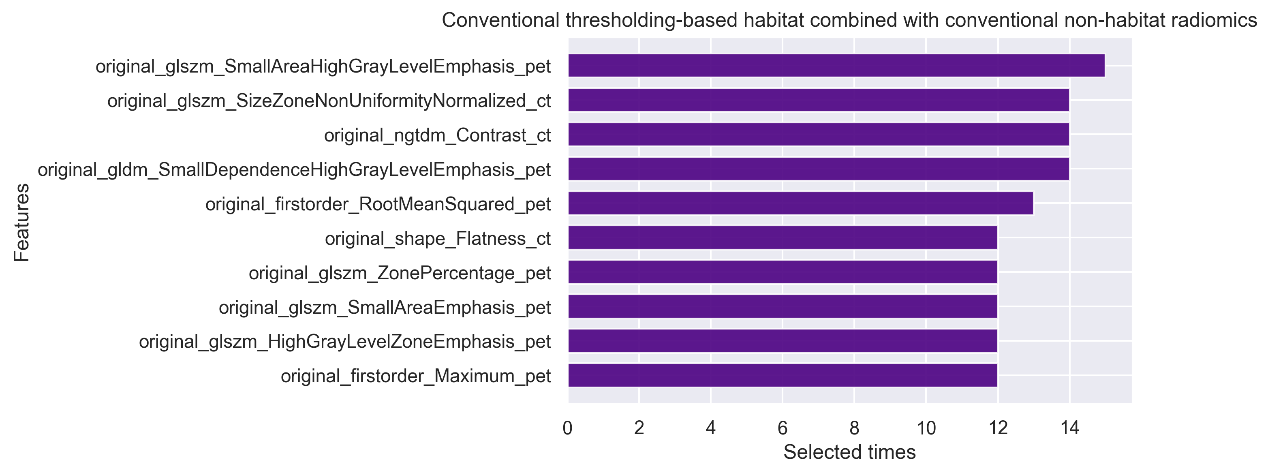
(c)


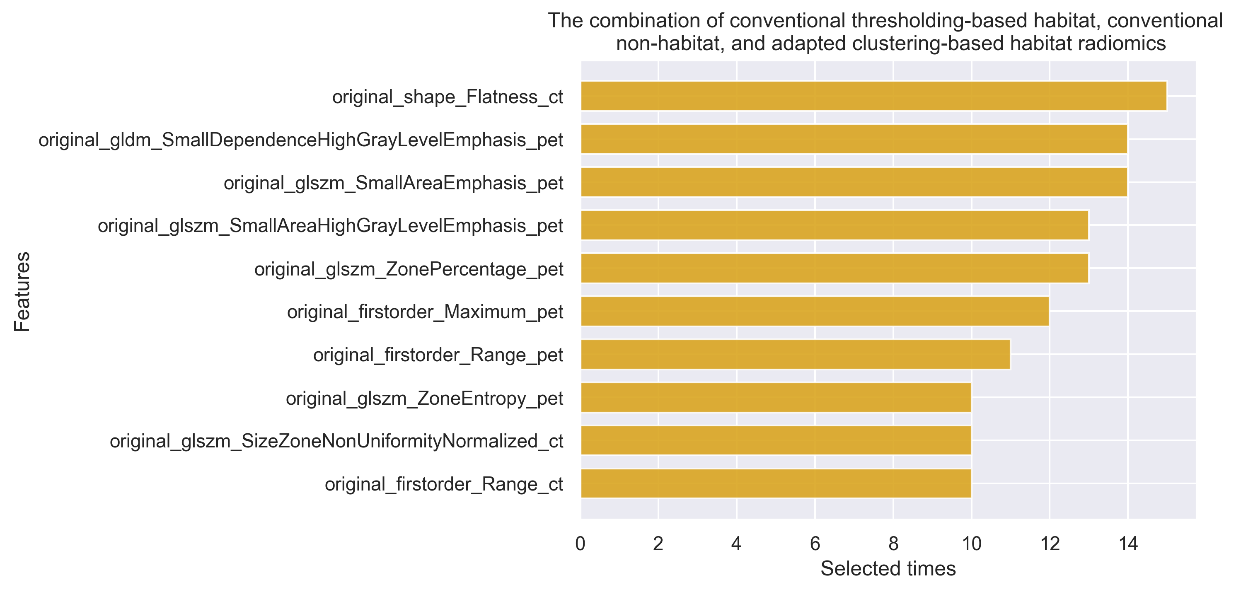
(d)

**Supplementary Figure 1** The top 10 selected features of (a) conventional thresholding-based, (b) conventional clustering-based, (c) conventional thresholding-based combined with nonhabitat, and (d) the combination of conventional thresholding-based, adapted clustering-based, and nohabiat methods


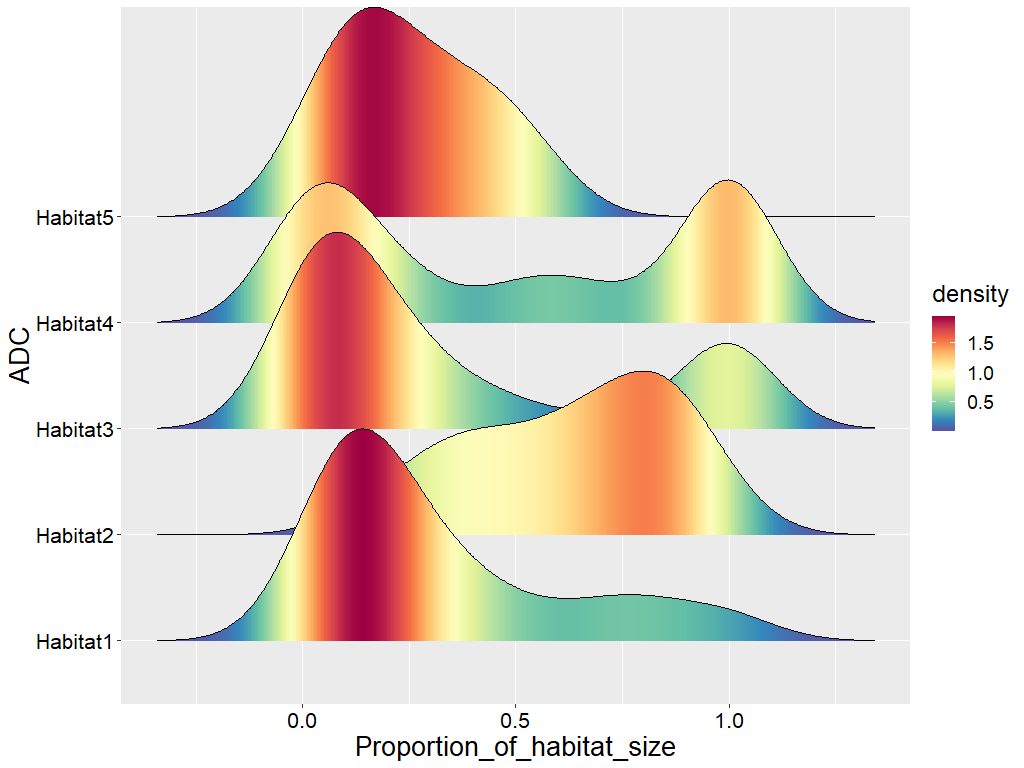

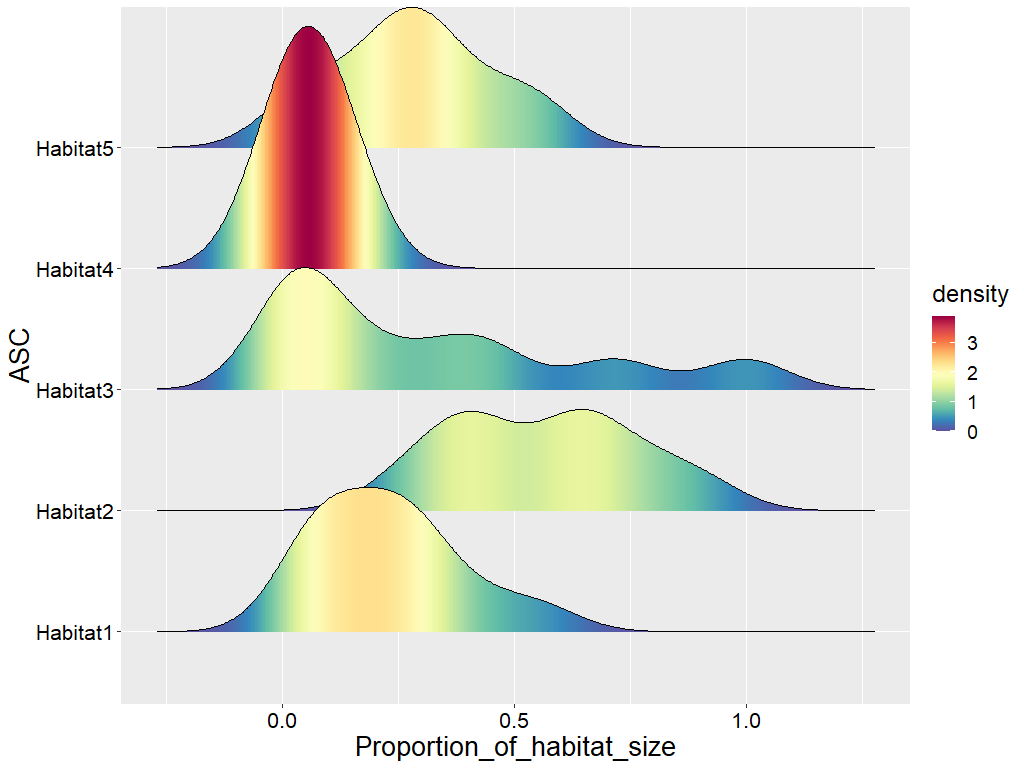


1. (b)


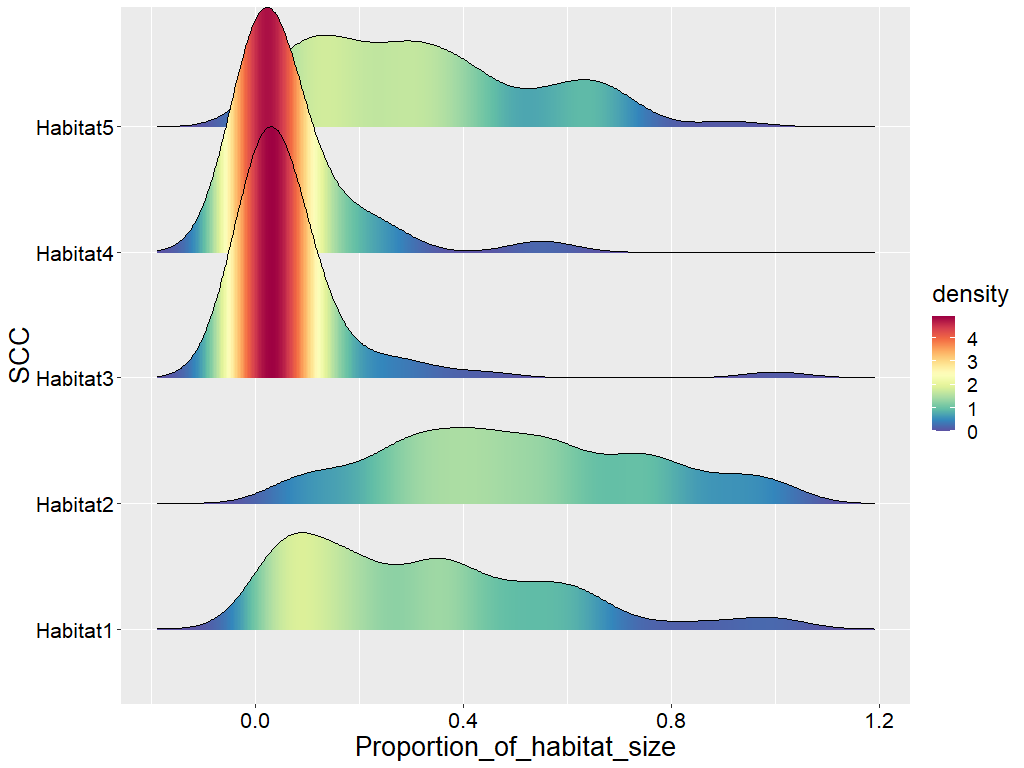

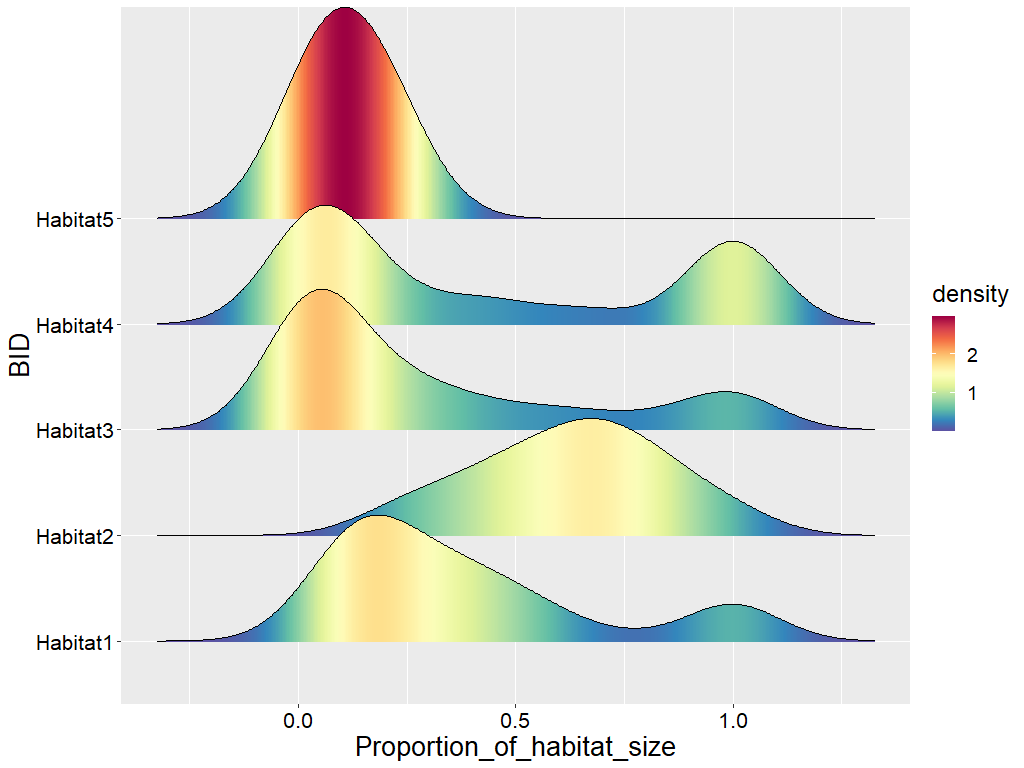


(c) (d)

**Supplementary Figure 2** The density of habitat size proportion for (a) ADC, (b) ASC, (c) SCC, and (d) BID

## Supplementary Tables

**Supplementary Table 1** The detail performances for specific combinations of feature selection method and classifier

| Habitat Method | Feature selection | Classifier | Five-fold train AUC | Five-fold train Acc. | Five-fold train prec. | Five-fold train sens. | Five-fold train spec. | Five-fold test AUC | Five-fold test acc. | Five-fold test prec. | Five-fold test sens. | Five-fold test spec. |
| --- | --- | --- | --- | --- | --- | --- | --- | --- | --- | --- | --- | --- |
| Conventional thresholding-based +  Non-habitat | SVM-RFE | Random forest | 0.9496 | 0.9438 | 0.962 | 0.9598 | 0.906 | 0.7343 | 0.7266 | 0.7928 | 0.838 | 0.4516 |
|  |  | Gradient bossting decision trees | 0.9553 | 0.97 | 0.9672 | 0.9914 | 0.9164 | 0.7231 | 0.7328 | 0.7906 | 0.8552 | 0.4306 |
|  |  | SVM | 0.8449 | 0.847 | 0.8456 | 0.9642 | 0.559 | 0.7142 | 0.7454 | 0.7844 | 0.8904 | 0.3854 |
|  |  | Logistic regression | 0.8053 | 0.8106 | 0.8196 | 0.9412 | 0.4896 | 0.7376 | 0.7358 | 0.7792 | 0.8812 | 0.3748 |
|  |  | Adaboost | 0.8103 | 0.8268 | 0.8196 | 0.9718 | 0.4704 | 0.7072 | 0.7294 | 0.7622 | 0.903 | 0.3012 |
|  |  | Bagging | 0.8244 | 0.8278 | 0.8386 | 0.9414 | 0.549 | 0.7316 | 0.7204 | 0.7672 | 0.8724 | 0.3432 |
|  | LASSO | Random forest | 0.9502 | 0.944 | 0.9574 | 0.9652 | 0.8924 | 0.7402 | 0.7296 | 0.8008 | 0.8242 | 0.4948 |
|  |  | Gradient bossting decision trees | 0.9444 | 0.9588 | 0.957 | 0.9872 | 0.8898 | 0.7336 | 0.7174 | 0.7932 | 0.8194 | 0.4602 |
|  |  | SVM | 0.8167 | 0.8268 | 0.8262 | 0.9608 | 0.4978 | 0.7119 | 0.7328 | 0.7718 | 0.8896 | 0.3428 |
|  |  | Logistic regression | 0.7755 | 0.7766 | 0.7848 | 0.9454 | 0.3598 | 0.7452 | 0.7486 | 0.7764 | 0.9176 | 0.3352 |
|  |  | Adaboost | 0.7871 | 0.7958 | 0.7946 | 0.962 | 0.387 | 0.7243 | 0.7388 | 0.7658 | 0.9168 | 0.3024 |
|  |  | Bagging | 0.8278 | 0.8238 | 0.8288 | 0.9508 | 0.5108 | 0.7405 | 0.7266 | 0.766 | 0.8948 | 0.3106 |
|  | Random forest | Random forest | 0.9362 | 0.9324 | 0.9374 | 0.9708 | 0.8388 | 0.7100 | 0.7326 | 0.7978 | 0.8378 | 0.472 |
|  |  | Gradient bossting decision trees | 0.8886 | 0.9154 | 0.913 | 0.9806 | 0.7564 | 0.6902 | 0.7112 | 0.7912 | 0.8108 | 0.4586 |
|  |  | SVM | 0.7624 | 0.8006 | 0.795 | 0.975 | 0.3712 | 0.6421 | 0.7296 | 0.764 | 0.9118 | 0.2782 |
|  |  | Logistic regression | 0.7247 | 0.7766 | 0.7746 | 0.9674 | 0.3064 | 0.6759 | 0.7296 | 0.7518 | 0.9252 | 0.248 |
|  |  | Adaboost | 0.7499 | 0.7928 | 0.7902 | 0.9662 | 0.3656 | 0.6625 | 0.6956 | 0.7282 | 0.9122 | 0.1604 |
|  |  | Bagging | 0.8077 | 0.8244 | 0.8216 | 0.9642 | 0.481 | 0.6984 | 0.7232 | 0.761 | 0.8904 | 0.3122 |
| Adapted clustering-based | SVM-RFE | Random forest | 0.9761 | 0.9592 | 0.9596 | 0.9844 | 0.8976 | 0.7279 | 0.7294 | 0.7806 | 0.8616 | 0.4064 |
|  |  | Gradient bossting decision trees | 0.9707 | 0.9568 | 0.9688 | 0.97 | 0.9244 | 0.6963 | 0.698 | 0.7788 | 0.8048 | 0.4416 |
|  |  | SVM | 0.8524 | 0.8444 | 0.8432 | 0.96 | 0.565 | 0.7268 | 0.7674 | 0.8 | 0.8976 | 0.4492 |
|  |  | Logistic regression | 0.8137 | 0.8184 | 0.8284 | 0.9412 | 0.522 | 0.7256 | 0.7042 | 0.7512 | 0.8704 | 0.3 |
|  |  | Adaboost | 0.8230 | 0.8126 | 0.8232 | 0.948 | 0.4876 | 0.7014 | 0.7198 | 0.7648 | 0.8742 | 0.341 |
|  |  | Bagging | 0.8078 | 0.805 | 0.8202 | 0.9324 | 0.4976 | 0.7201 | 0.7234 | 0.7536 | 0.9064 | 0.2778 |
|  | LASSO | Random forest | 0.9789 | 0.9632 | 0.9662 | 0.9822 | 0.9164 | 0.7498 | 0.7514 | 0.8076 | 0.8528 | 0.5046 |
|  |  | Gradient bossting decision trees | 0.93 | 0.919 | 0.9172 | 0.9754 | 0.7826 | 0.7264 | 0.7324 | 0.7938 | 0.8388 | 0.4712 |
|  |  | SVM | 0.8439 | 0.8334 | 0.858 | 0.9178 | 0.6292 | 0.7198 | 0.7258 | 0.7862 | 0.8378 | 0.4496 |
|  |  | Logistic regression | 0.7851 | 0.779 | 0.7838 | 0.951 | 0.3628 | 0.7451 | 0.7234 | 0.7494 | 0.9152 | 0.2578 |
|  |  | Adaboost | 0.7904 | 0.7798 | 0.787 | 0.9458 | 0.3792 | 0.7314 | 0.6884 | 0.7284 | 0.8882 | 0.2042 |
|  |  | Bagging | 0.835 | 0.8106 | 0.8094 | 0.9598 | 0.4486 | 0.7429 | 0.7294 | 0.7614 | 0.9022 | 0.313 |
|  | Random forest | Random forest | 0.9762 | 0.952 | 0.9518 | 0.9832 | 0.876 | 0.7418 | 0.745 | 0.7884 | 0.875 | 0.4298 |
|  |  | Gradient bossting decision trees | 0.8457 | 0.8254 | 0.8338 | 0.9464 | 0.532 | 0.7339 | 0.7514 | 0.7754 | 0.9158 | 0.3556 |
|  |  | SVM | 0.8184 | 0.7978 | 0.7986 | 0.9568 | 0.4136 | 0.7126 | 0.742 | 0.7622 | 0.9248 | 0.3018 |
|  |  | Logistic regression | 0.773 | 0.7682 | 0.7682 | 0.9632 | 0.2954 | 0.740250605 | 0.7484 | 0.758 | 0.9472 | 0.2686 |
|  |  | Adaboost | 0.7815 | 0.7642 | 0.7726 | 0.949 | 0.3172 | 0.7125 | 0.7072 | 0.741 | 0.9016 | 0.2358 |
|  |  | Bagging | 0.794 | 0.7932 | 0.8012 | 0.9454 | 0.4244 | 0.7305 | 0.695 | 0.739 | 0.8796 | 0.248 |
| Conventional thresholding-based | SVM-RFE | Random forest | 0.9568 | 0.9368 | 0.9348 | 0.9814 | 0.8278 | 0.7262 | 0.7318 | 0.7736 | 0.8816 | 0.3658 |
|  |  | Gradient bossting decision trees | 0.9632 | 0.968 | 0.9652 | 0.9914 | 0.9112 | 0.709 | 0.704 | 0.7688 | 0.833 | 0.3864 |
|  |  | SVM | 0.8484 | 0.8444 | 0.8438 | 0.9594 | 0.562 | 0.719 | 0.7134 | 0.7528 | 0.8898 | 0.279 |
|  |  | Logistic regression | 0.7954 | 0.8054 | 0.8096 | 0.9496 | 0.4516 | 0.727 | 0.7322 | 0.7654 | 0.9028 | 0.314 |
|  |  | Adaboost | 0.8071 | 0.8318 | 0.8314 | 0.9584 | 0.5218 | 0.6803 | 0.7164 | 0.7598 | 0.8808 | 0.311 |
|  |  | Bagging | 0.8129 | 0.8168 | 0.8388 | 0.9212 | 0.5622 | 0.7266 | 0.735 | 0.7816 | 0.8716 | 0.397 |
|  | LASSO | Random forest | 0.9447 | 0.9354 | 0.9444 | 0.9672 | 0.858 | 0.7308 | 0.7382 | 0.7926 | 0.8548 | 0.4508 |
|  |  | Gradient bossting decision trees | 0.9438 | 0.9494 | 0.9708 | 0.9584 | 0.9272 | 0.7032 | 0.6756 | 0.768 | 0.7806 | 0.4218 |
|  |  | SVM | 0.7946 | 0.8192 | 0.8216 | 0.955 | 0.4872 | 0.7047 | 0.7352 | 0.7678 | 0.9028 | 0.321 |
|  |  | Logistic regression | 0.7536 | 0.7718 | 0.7724 | 0.9628 | 0.3036 | 0.7258 | 0.7474 | 0.7578 | 0.9468 | 0.259 |
|  |  | Adaboost | 0.7627 | 0.7852 | 0.7924 | 0.9488 | 0.3852 | 0.715627278 | 0.7382 | 0.7612 | 0.92 | 0.289 |
|  |  | Bagging | 0.8194 | 0.82 | 0.8178 | 0.9628 | 0.4704 | 0.7221 | 0.7476 | 0.7688 | 0.9248 | 0.3122 |
|  | Random forest | Random forest | 0.9508 | 0.9422 | 0.95 | 0.9704 | 0.8738 | 0.7022 | 0.701 | 0.7734 | 0.82 | 0.4086 |
|  |  | Gradient bossting decision trees | 0.8514 | 0.8528 | 0.8598 | 0.9618 | 0.5872 | 0.6875 | 0.7166 | 0.7708 | 0.8634 | 0.353 |
|  |  | SVM | 0.7594 | 0.7796 | 0.771 | 0.9824 | 0.2822 | 0.6786 | 0.7258 | 0.7424 | 0.9434 | 0.1956 |
|  |  | Logistic regression | 0.7242 | 0.7796 | 0.7724 | 0.9782 | 0.293 | 0.7021 | 0.7568 | 0.7608 | 0.9606 | 0.2592 |
|  |  | Adaboost | 0.7417 | 0.7688 | 0.7672 | 0.9704 | 0.2746 | 0.6922 | 0.7258 | 0.7504 | 0.9248 | 0.2356 |
|  |  | Bagging | 0.7996 | 0.821 | 0.8268 | 0.9464 | 0.5132 | 0.7083 | 0.7258 | 0.7598 | 0.8992 | 0.3024 |
| Adapted clustering-based + Non-habitat | SVM-RFE | Random forest | 0.9856 | 0.9804 | 0.9796 | 0.9934 | 0.9488 | 0.7451 | 0.7564 | 0.8054 | 0.8678 | 0.4826 |
|  |  | Gradient bossting decision trees | 0.9652 | 0.9624 | 0.9608 | 0.9882 | 0.9004 | 0.7337 | 0.747 | 0.7942 | 0.8676 | 0.4504 |
|  |  | SVM | 0.8727 | 0.8586 | 0.8638 | 0.9516 | 0.6318 | 0.7251 | 0.7532 | 0.7908 | 0.8896 | 0.4186 |
|  |  | Logistic regression | 0.8451 | 0.8366 | 0.8432 | 0.9474 | 0.5676 | 0.7396 | 0.7624 | 0.7898 | 0.9074 | 0.4092 |
|  |  | Adaboost | 0.8698 | 0.8618 | 0.8726 | 0.9472 | 0.6538 | 0.6917 | 0.7156 | 0.7686 | 0.8584 | 0.3636 |
|  |  | Bagging | 0.8308 | 0.8298 | 0.8432 | 0.9352 | 0.5728 | 0.7256 | 0.75 | 0.7756 | 0.916 | 0.3432 |
|  | LASSO | Random forest | 0.981 | 0.975 | 0.973 | 0.9924 | 0.9328 | 0.7478 | 0.722 | 0.7886 | 0.8326 | 0.4492 |
|  |  | Gradient bossting decision trees | 0.962 | 0.9576 | 0.9644 | 0.977 | 0.9112 | 0.7420 | 0.747 | 0.8026 | 0.8546 | 0.4836 |
|  |  | SVM | 0.8479 | 0.8352 | 0.8336 | 0.9602 | 0.5298 | 0.7491 | 0.7596 | 0.772 | 0.9386 | 0.3228 |
|  |  | Logistic regression | 0.7989 | 0.786 | 0.7864 | 0.9626 | 0.355 | 0.7471 | 0.7344 | 0.7482 | 0.9428 | 0.2258 |
|  |  | Adaboost | 0.8033 | 0.7928 | 0.8112 | 0.9274 | 0.4656 | 0.7334 | 0.7282 | 0.762 | 0.8984 | 0.3102 |
|  |  | Bagging | 0.8632 | 0.85 | 0.8486 | 0.9604 | 0.5806 | 0.7537 | 0.7468 | 0.7746 | 0.9074 | 0.354 |
|  | Random forest | Random forest | 0.9476 | 0.932 | 0.9228 | 0.988 | 0.7952 | 0.7325 | 0.7376 | 0.7768 | 0.8856 | 0.376 |
|  |  | Gradient bossting decision trees | 0.8382 | 0.8376 | 0.8318 | 0.9746 | 0.503 | 0.7251 | 0.7376 | 0.7672 | 0.903 | 0.332 |
|  |  | SVM | 0.7927 | 0.8048 | 0.8056 | 0.9582 | 0.43 | 0.6985 | 0.747 | 0.7768 | 0.9074 | 0.3546 |
|  |  | Logistic regression | 0.7728 | 0.7802 | 0.7826 | 0.9572 | 0.3498 | 0.7478 | 0.7562 | 0.7674 | 0.9474 | 0.2908 |
|  |  | Adaboost | 0.7828 | 0.7858 | 0.7914 | 0.9504 | 0.3846 | 0.7176 | 0.7376 | 0.7644 | 0.9162 | 0.3006 |
|  |  | Bagging | 0.8154 | 0.8116 | 0.8108 | 0.9592 | 0.4512 | 0.7351 | 0.722 | 0.7514 | 0.912 | 0.2596 |
| Adapted clustering-based + Non-habitat + Conventional thresholding-based | SVM-RFE | Random forest | 0.9373 | 0.935 | 0.9346 | 0.9772 | 0.8304 | 0.7249 | 0.7514 | 0.8094 | 0.851 | 0.5052 |
|  |  | Gradient bossting decision trees | 0.9277 | 0.9458 | 0.9522 | 0.9728 | 0.8792 | 0.7253 | 0.7328 | 0.8066 | 0.82 | 0.5158 |
|  |  | SVM | 0.8458 | 0.8452 | 0.8454 | 0.9608 | 0.5622 | 0.7204 | 0.7514 | 0.7802 | 0.9076 | 0.3642 |
|  |  | Logistic regression | 0.8099 | 0.83 | 0.8344 | 0.9498 | 0.5354 | 0.7237 | 0.7296 | 0.7678 | 0.8902 | 0.3334 |
|  |  | Adaboost | 0.7946 | 0.8228 | 0.8162 | 0.9718 | 0.4576 | 0.684 | 0.7202 | 0.7468 | 0.9158 | 0.235 |
|  |  | Bagging | 0.8326 | 0.8352 | 0.8428 | 0.9466 | 0.5614 | 0.7251 | 0.7358 | 0.7736 | 0.89 | 0.3556 |
|  | LASSO | Random forest | 0.939 | 0.9434 | 0.9416 | 0.9814 | 0.8492 | 0.742 | 0.7544 | 0.807 | 0.8594 | 0.4948 |
|  |  | Gradient bossting decision trees | 0.9257 | 0.9502 | 0.9476 | 0.9848 | 0.8656 | 0.7262 | 0.7326 | 0.7892 | 0.8506 | 0.4408 |
|  |  | SVM | 0.8306 | 0.8448 | 0.8486 | 0.9532 | 0.5784 | 0.7166 | 0.739 | 0.7744 | 0.894 | 0.3526 |
|  |  | Logistic regression | 0.7886 | 0.8108 | 0.8228 | 0.9356 | 0.5028 | 0.7193 | 0.7514 | 0.7836 | 0.8996 | 0.3876 |
|  |  | Adaboost | 0.7845 | 0.812 | 0.8184 | 0.9486 | 0.4762 | 0.6918 | 0.72 | 0.7516 | 0.9028 | 0.2692 |
|  |  | Bagging | 0.8419 | 0.8486 | 0.8584 | 0.9456 | 0.6098 | 0.737332131 | 0.7516 | 0.785 | 0.8996 | 0.3884 |
|  | Random forest | Random forest | 0.9055 | 0.903 | 0.8916 | 0.9848 | 0.7014 | 0.7116 | 0.7112 | 0.767 | 0.851 | 0.3654 |
|  |  | Gradient bossting decision trees | 0.8492 | 0.872 | 0.872 | 0.9718 | 0.6268 | 0.6962 | 0.6894 | 0.7502 | 0.8424 | 0.3116 |
|  |  | SVM | 0.8177 | 0.8252 | 0.8298 | 0.95 | 0.5186 | 0.6909 | 0.7516 | 0.7914 | 0.8818 | 0.4302 |
|  |  | Logistic regression | 0.7386 | 0.7614 | 0.7826 | 0.9234 | 0.3624 | 0.7119 | 0.714 | 0.755 | 0.891 | 0.2826 |
|  |  | Adaboost | 0.7491 | 0.76 | 0.7624 | 0.963 | 0.2606 | 0.6965 | 0.711 | 0.7324 | 0.9338 | 0.1616 |
|  |  | Bagging | 0.816 | 0.8262 | 0.8398 | 0.9346 | 0.559 | 0.7161 | 0.7048 | 0.7514 | 0.8728 | 0.2908 |
| Conventional clustering-based | SVM-RFE | Random forest | 0.9885 | 0.9714 | 0.9808 | 0.98 | 0.9506 | 0.6772 | 0.716 | 0.7968 | 0.8172 | 0.4642 |
|  |  | Gradient bossting decision trees | 0.9538 | 0.94 | 0.9438 | 0.9744 | 0.8556 | 0.6475 | 0.707 | 0.7748 | 0.8356 | 0.3892 |
|  |  | SVM | 0.8291 | 0.8296 | 0.823 | 0.9688 | 0.4892 | 0.6439 | 0.719 | 0.7618 | 0.8836 | 0.3128 |
|  |  | Logistic regression | 0.7847 | 0.7964 | 0.7976 | 0.9568 | 0.4048 | 0.6728 | 0.7286 | 0.7532 | 0.9194 | 0.2598 |
|  |  | Adaboost | 0.8461 | 0.8374 | 0.8548 | 0.932 | 0.6058 | 0.6716 | 0.7064 | 0.7678 | 0.8438 | 0.3702 |
|  |  | Bagging | 0.7884 | 0.7938 | 0.7972 | 0.9532 | 0.4048 | 0.6774 | 0.7222 | 0.7546 | 0.9104 | 0.259 |
|  | LASSO | Random forest | 0.9345 | 0.9118 | 0.931 | 0.9454 | 0.8288 | 0.672 | 0.6908 | 0.7776 | 0.7988 | 0.4222 |
|  |  | Gradient bossting decision trees | 0.8193 | 0.8256 | 0.841 | 0.9334 | 0.5622 | 0.6788 | 0.7256 | 0.7718 | 0.8758 | 0.3568 |
|  |  | SVM | 0.7384 | 0.7824 | 0.7788 | 0.969 | 0.3262 | 0.6042 | 0.7448 | 0.7622 | 0.9338 | 0.2808 |
|  |  | Logistic regression | 0.7134 | 0.7692 | 0.777 | 0.9468 | 0.3346 | 0.6436 | 0.7288 | 0.756 | 0.9194 | 0.2586 |
|  |  | Adaboost | 0.7269 | 0.7682 | 0.7668 | 0.97 | 0.2746 | 0.644 | 0.7286 | 0.7422 | 0.946 | 0.1954 |
|  |  | Bagging | 0.8004 | 0.8004 | 0.8098 | 0.9446 | 0.4488 | 0.6448 | 0.6876 | 0.7378 | 0.8746 | 0.2258 |
|  | Random forest | Random forest | 0.9574 | 0.94 | 0.9526 | 0.963 | 0.8832 | 0.66385 | 0.7096 | 0.7922 | 0.8088 | 0.4662 |
|  |  | Gradient bossting decision trees | 0.9159 | 0.911 | 0.9096 | 0.9754 | 0.7528 | 0.6615 | 0.7068 | 0.7712 | 0.8486 | 0.3556 |
|  |  | SVM | 0.7707 | 0.8002 | 0.7982 | 0.9632 | 0.4022 | 0.6107 | 0.7224 | 0.7578 | 0.898 | 0.2926 |
|  |  | Logistic regression | 0.7228 | 0.7692 | 0.7662 | 0.9724 | 0.272 | 0.6581 | 0.735 | 0.7488 | 0.9464 | 0.2164 |
|  |  | Adaboost | 0.7821 | 0.7988 | 0.8074 | 0.9444 | 0.4434 | 0.6834 | 0.7508 | 0.7852 | 0.9012 | 0.379 |
|  |  | Bagging | 0.7937 | 0.8042 | 0.8078 | 0.951 | 0.4456 | 0.6638 | 0.7382 | 0.7604 | 0.9246 | 0.2808 |
| Non-habitat | SVM-RFE | Random forest | 0.9934 | 0.9684 | 0.9632 | 0.9942 | 0.9052 | 0.7169 | 0.7128 | 0.7866 | 0.8222 | 0.4468 |
|  |  | Gradient bossting decision trees | 0.9719 | 0.945 | 0.9438 | 0.9832 | 0.8508 | 0.6864 | 0.6844 | 0.7694 | 0.7954 | 0.4134 |
|  |  | SVM | 0.8276 | 0.817 | 0.8392 | 0.9276 | 0.5456 | 0.6637 | 0.6848 | 0.7562 | 0.8266 | 0.3376 |
|  |  | Logistic regression | 0.8046 | 0.7918 | 0.7944 | 0.9588 | 0.3822 | 0.7019 | 0.7284 | 0.7654 | 0.8978 | 0.318 |
|  |  | Adaboost | 0.8695 | 0.8366 | 0.8398 | 0.959 | 0.5374 | 0.6865 | 0.7382 | 0.78 | 0.8844 | 0.3826 |
|  |  | Bagging | 0.7916 | 0.777 | 0.7982 | 0.9222 | 0.4208 | 0.6947 | 0.7094 | 0.757 | 0.8756 | 0.3048 |
|  | LASSO | Random forest | 0.9929 | 0.9734 | 0.98 | 0.9822 | 0.951 | 0.7146 | 0.6718 | 0.799 | 0.7246 | 0.5428 |
|  |  | Gradient bossting decision trees | 0.9576 | 0.9328 | 0.934 | 0.9778 | 0.824 | 0.6777 | 0.6844 | 0.7766 | 0.7822 | 0.4452 |
|  |  | SVM | 0.867 | 0.8358 | 0.844 | 0.9456 | 0.5678 | 0.6756 | 0.7 | 0.7636 | 0.8402 | 0.3592 |
|  |  | Logistic regression | 0.8374 | 0.813 | 0.8182 | 0.9476 | 0.4834 | 0.7211 | 0.7444 | 0.78 | 0.8934 | 0.3818 |
|  |  | Adaboost | 0.8956 | 0.847 | 0.8518 | 0.9544 | 0.584 | 0.6915 | 0.7158 | 0.7636 | 0.8712 | 0.3356 |
|  |  | Bagging | 0.82 | 0.7932 | 0.8058 | 0.9378 | 0.4394 | 0.7002 | 0.7034 | 0.7554 | 0.8666 | 0.3066 |
|  | Random forest | Random forest | 0.9926 | 0.9764 | 0.9862 | 0.9808 | 0.9648 | 0.7033 | 0.6784 | 0.7822 | 0.76 | 0.4784 |
|  |  | Gradient bossting decision trees | 0.9168 | 0.8952 | 0.8934 | 0.9754 | 0.6986 | 0.69217 | 0.6846 | 0.7732 | 0.791 | 0.4228 |
|  |  | SVM | 0.8118 | 0.7924 | 0.798 | 0.95 | 0.407 | 0.6579 | 0.7316 | 0.7784 | 0.88 | 0.3726 |
|  |  | Logistic regression | 0.7788 | 0.7722 | 0.7764 | 0.9564 | 0.3208 | 0.7080 | 0.7382 | 0.7656 | 0.9154 | 0.3048 |
|  |  | Adaboost | 0.8184 | 0.788 | 0.788 | 0.9622 | 0.3608 | 0.6738 | 0.7196 | 0.7562 | 0.9022 | 0.2748 |
|  |  | Bagging | 0.8038 | 0.7786 | 0.7766 | 0.9666 | 0.3178 | 0.7212 | 0.7256 | 0.74 | 0.9468 | 0.1848 |

# Supplementary Information of Radiomics features

1. First-order features
   1. Energy
      $energy=\sum_{i=1}^{N_{p}} {(X\left( i \right)+c)}^{2}$
   2. Total energy
      $total energy=V_{voxel}\sum_{i=1}^{N_{p}} {(X\left( i \right)+c)}^{2}$
   3. Entropy

$$entropy=-\sum_{i=1}^{N_{g}} p\left( i \right){log}_{2}\left( p\left( i \right)+\epsilon\right)$$

Where $\epsilon$ is approximate to 2.2 * 10e-16. In IBSI, it was defined as Itensity Histogram Entropy

- 1. Minimum

$$minimum=\min\left( X \right)$$

The minimum gray level intensity within the VOI.

- 1. 10^th^ percentile
  $The 10th percentile of X$
  2. 90^th^ percentile
     $The 90th percentile of X$
  3. Maximum

$$maximum=max(X)$$

The maximum gray level intensity within the VOI.

- 1. Mean
  $mean=\frac{1}{N_{p}}\sum_{i=1}^{N_{p}} X(i)$
  2. Median
     $The median gray level intensity within the VOI.$
  3. Interquartile range
     $interquartile range=P_{75}-P_{25}$
  4. Range
     $range=\max\left( X \right)-min(X)$
  5. Mean absolute deviation (MAD)
     $MAD=\frac{1}{N_{p}}$
  6. Robust mean absolute deviation (rMAD)
     $rMAD=\frac{1}{N_{10-90}}\sum_{i=1}^{N_{10-90}} |X_{10-90}(i)-\overline{X}_{10-90}|$
  7. Standard deviation
     $standard deviation=\sqrt{\frac{1}{N_{p}}{\sum_{i=1}^{N_{p}} (X\left( i \right)-\overline{X})}^{2}}$
  8. Root mean square (RMS)
     $RMS=\sqrt{\frac{1}{N_{p}}{\sum_{i=1}^{N_{p}} X\left( i \right)}^{2}}$
  9. Skewness
     $skewness=\frac{{\frac{1}{N_{p}}\sum_{i=1}^{N_{p}} (X\left( i \right)-\overline{X})}^{3}}{\sqrt{\frac{1}{N_{p}}{\sum_{i=1}^{N_{p}} (X\left( i \right)-\overline{X})}^{2}}}$
  10. Kurtosis
      $kurtosis=\frac{{\frac{1}{N_{p}}\sum_{i=1}^{N_{p}} (X\left( i \right)-\overline{X})}^{4}}{{(\frac{1}{N_{p}}{\sum_{i=1}^{N_{p}} (X\left( i \right)-\overline{X})}^{2})}^{2}}$
  11. Kurtosis
      $variance={\frac{1}{N_{p}}\sum_{i=1}^{N_{p}} (X\left( i \right)-\overline{X})}^{2}$
  12. Kurtosis

$$uniformity={\frac{1}{N_{p}}\sum_{i=1}^{N_{p}} (X\left( i \right)-\overline{X})}^{2}$$

1. Shape features
   1. Mesh volume
      $V_{i}=\frac{O_{ai}\cdot(O_{bi}\times O_{ci})}{6}$
      $V={\sum_{i=1}^{N_{f}} V_{i}}$
   2. Voxel volume

$$V_{voxel}=\sum_{i=1}^{N_{v}} V_{k}$$

V_k_ is equal to the volume for a single voxel.

- 1. Surface area
  $A_{i}=\frac{1}{2}|a_{i}b_{i}\times a_{i}c_{i}|$
  $A=\sum_{i=1}^{N_{v}} A_{i}$
  2. Surface area to volume ratio
     $surface to volume ratio=\frac{A}{V}$
  3. Sphericity
     $sphericity=\frac{\sqrt[3]{36\pi V^{2}}}{A}$
  4. Compactness 1
     $compactness 1=\frac{V}{\sqrt{\pi A^{3}}}$
  5. Compactness 2
     $compactness 2=36\pi\frac{V^{2}}{A^{2}}$
  6. Spherical disproportion
     $spherical disproportion=\frac{A}{\sqrt[3]{36\pi V^{2}}}$
  7. Maximum 3D diameter
     $The largest pairwise Euclidean distance between tumor surface mesh vertices.$
  8. Maximum 2D diameter (Slice)
     $The largest pairwise Euclidean distance between tumor surface mesh vertices in$
     $the axial plane.$
  9. Maximum 2D diameter (Column)
     $The largest pairwise Euclidean distance between tumor surface mesh vertices in$
     $the coronal plane.$
  10. Maximum 2d diameter (Row)
      $The largest pairwise Euclidean distance between tumor surface mesh vertices in$
      $the sagittal plane.$
  11. Major axis length

$$major axis=4\sqrt{\lambda_{major}}$$

The $\lambda_{major}$ is the largest principal component to represent the largest axis length of the VOI-enclosing ellipsoid.

- 1. Minor axis length

$$minor axis=4\sqrt{\lambda_{minor}}$$

The $\lambda_{minor}$ is the second-largest principal component to represent the second-largest axis length of the VOI-enclosing ellipsoid.

- 1. Least axis length

$$least axis=4\sqrt{\lambda_{least}}$$

The $\lambda_{least}$ is the smallest principal component to represent the smallest axis length of the VOI-enclosing ellipsoid.

- 1. Elongation
  $elongation= \sqrt{\frac{\lambda_{least}}{\lambda_{major}}}$
  2. Flatness

$$flatness= \sqrt{\frac{\lambda_{least}}{\lambda_{minor}}}$$

1. Gray level co-occurrence matrix (GLCM) features
   1. Autocorrelation
      $autocorrelation=\sum_{i=1}^{N_{g}} \sum_{j=1}^{N_{g}} p(i,j)ij$
   2. Joint average
      $joint average=\sum_{i=1}^{N_{g}} \sum_{j=1}^{N_{g}} p(i,j)i$
   3. Cluster Prominence
      $cluster prominence=\sum_{i=1}^{N_{g}} \sum_{j=1}^{N_{g}} {(i+j-\mu_{x}-\mu_{y})}^{4}p(i,j)$
   4. Cluster shade
      $cluster shade=\sum_{i=1}^{N_{g}} \sum_{j=1}^{N_{g}} {(i+j-\mu_{x}-\mu_{y})}^{3}p(i,j)$
   5. Cluster tendency
      $cluster tendency=\sum_{i=1}^{N_{g}} \sum_{j=1}^{N_{g}} {(i+j-\mu_{x}-\mu_{y})}^{2}p(i,j)$
   6. Contrast
      $contrast=\sum_{i=1}^{N_{g}} \sum_{j=1}^{N_{g}} {(i-j)}^{2}p(i,j)$
   7. Correlation
      $correlation=\frac{\sum_{i=1}^{N_{g}} \sum_{j=1}^{N_{g}} p(i,j)ij-\mu_{x}-\mu_{y}}{\sigma_{x}(i)\sigma_{y}(j)}$
   8. Difference average
      $difference average=\sum_{k=0}^{N_{g}-1} kp_{x-y}(k)$
   9. Difference entropy
      $difference entropy=\sum_{k=0}^{N_{g}-1} p_{x-y}(k){log}_{2}(p_{x-y}\left( k \right)+\epsilon)$
   10. Difference variance
       $difference variance=\sum_{k=0}^{N_{g}-1} \left( k-DA \right)^{2}p_{x-y}(k)$
   11. Joint energy
       $joint energy=\sum_{i=1}^{N_{g}} \sum_{j=1}^{N_{g}} {p(i,j)}^{2}$
   12. Joint entropy
       $joint entropy=-\sum_{i=1}^{N_{g}} \sum_{j=1}^{N_{g}} p(i,j){log}_{2}(p_{x-y}\left( k \right)+\epsilon)$
   13. Inverse difference moment
       $inverse difference moment=\sum_{k=0}^{N_{g}-1} \frac{p_{x-y}(k)}{1+k^{2}}$
   14. Maximal correlation coefficient
       $maximal correlation coefficient=\sqrt{the second largest eigenvalue of Q}$
       $Q\left( i,j \right)=\sum_{k=0}^{N_{g}} \frac{p(i,k)p(j,k)}{p_{x}(i)p_{y}(k)}$
   15. Inverse difference moment normalized
       $inverse difference moment normalized=\sum_{k=0}^{N_{g}-1} \frac{p_{x-y}(k)}{1+(\frac{k^{2}}{N_{g}^{2}})}$
   16. Inverse difference
       $inverse difference normalized=\sum_{k=0}^{N_{g}-1} \frac{p_{x-y}(k)}{1+k}$
   17. Inverse difference normalized
       $inverse difference moment normalized=\sum_{k=0}^{N_{g}-1} \frac{p_{x-y}(k)}{1+(\frac{k}{N_{g}})}$
   18. Inverse variance
       $inverse variance=\sum_{k=0}^{N_{g}-1} \frac{p_{x-y}(k)}{k^{2}}$
   19. Maximum probability
       $maximum probability=max(p(i,j))$
   20. Sum average
       $sum average=\sum_{k=2}^{2N_{g}} p_{x+y}(k)k$
   21. Sum entropy
       $sum entropy=\sum_{k=2}^{2N_{g}} p_{x+y}(k){log}_{2}(p_{x+y}\left( k \right)+\epsilon)$
   22. Sum of squares

$sum entropy=\sum_{i=1}^{N_{g}} \sum_{j=1}^{N_{g}} p_{x+y}(k){log}_{2}(p_{x+y}\left( k \right)+\epsilon)$

2. Gray level size zone matrix features
   1. Small area emphasis
      $small area emphasis=\frac{\sum_{i=1}^{N_{g}} \sum_{j=1}^{N_{s}} \frac{P(i,j)}{j^{2}}}{N_{z}}$
   2. Large area emphasis
      $large area emphasis=\frac{\sum_{i=1}^{N_{g}} \sum_{j=1}^{N_{s}} {P(i,j)j}^{2}}{N_{z}}$
   3. Gray level non-uniformity
      $gray level nonuniformity=\frac{\sum_{i=1}^{N_{g}} (\sum_{j=1}^{N_{s}} {P(i,j))}^{2}}{N_{z}}$
   4. Gray level non-uniformity normalized
      $gray level nonuniformity normalized=\frac{\sum_{i=1}^{N_{g}} (\sum_{j=1}^{N_{s}} {P(i,j))}^{2}}{{N_{z}}^{2}}$
   5. Size-zone non-uniformity
      $size zone nonuniformity=\frac{\sum_{i=1}^{N_{s}} (\sum_{j=1}^{N_{g}} {P(i,j))}^{2}}{N_{z}}$
   6. Size-zone non-uniformity normalized
      $size zone nonuniformity normalized=\frac{\sum_{i=1}^{N_{s}} (\sum_{j=1}^{N_{g}} {P(i,j))}^{2}}{{N_{z}}^{2}}$
   7. Zone percentage
      $zone percentage=\frac{N_{z}}{N_{p}}$
   8. Gray level variance
      $gray level variance=\sum_{i=1}^{N_{g}} \sum_{j=1}^{N_{s}} p(i,j){(i-u)}^{2}$
      $u=\sum_{i=1}^{N_{g}} \sum_{j=1}^{N_{s}} p\left( i,j \right)i$
   9. Zone variance
      $zone variance=\sum_{i=1}^{N_{g}} \sum_{j=1}^{N_{s}} p(i,j){(j-u)}^{2}$
      $u=\sum_{i=1}^{N_{g}} \sum_{j=1}^{N_{s}} p\left( i,j \right)j$
   10. Zone entropy
       $zone \mathrm{entropy}=-\sum_{i=1}^{N_{g}} \sum_{j=1}^{N_{s}} p\left( i,j \right){log}_{2}\left( p\left( i,j \right)+\epsilon\right)$
       $where \epsilon is approximate to 2.2*10e-16.$
   11. Low gray level zone emphasis
       $low gray level zone emphasis= \frac{\sum_{i=1}^{N_{g}} \sum_{j=1}^{N_{s}} \frac{P\left( i,j \right)}{i^{2}}}{N_{z}}$
   12. High gray level zone emphasis
       $high gray level zone emphasis= \frac{\sum_{i=1}^{N_{g}} \sum_{j=1}^{N_{s}} P\left( i,j \right)i^{2}}{N_{z}}$
   13. Small area low gray level emphasis
       $small area low gray level zone emphasis= \frac{\sum_{i=1}^{N_{g}} \sum_{j=1}^{N_{s}} \frac{P\left( i,j \right)}{i^{2}j^{2}}}{N_{z}}$
   14. Small area high gray level emphasis
       $small area high gray level zone emphasis= \frac{\sum_{i=1}^{N_{g}} \sum_{j=1}^{N_{s}} \frac{P\left( i,j \right)i^{2}}{j^{2}}}{N_{z}}$
   15. Large area low gray level emphasis
       $large area low gray level zone emphasis= \frac{\sum_{i=1}^{N_{g}} \sum_{j=1}^{N_{s}} \frac{P\left( i,j \right)j^{2}}{i^{2}}}{N_{z}}$
   16. Large area high gray level emphasis

$large area high gray level zone emphasis= \frac{\sum_{i=1}^{N_{g}} \sum_{j=1}^{N_{s}} P\left( i,j \right)i^{2}j^{2}}{N_{z}}$

3. Gray level run length matrix features
   1. Short run emphasis
      $short run emphasis= \frac{\sum_{i=1}^{N_{g}} \sum_{j=1}^{N_{s}} \frac{P\left( i,j|\theta\right)}{j^{2}}}{N_{r}(\theta)}$
   2. Long run emphasis
      $long run emphasis=\frac{\sum_{i=1}^{N_{g}} \sum_{j=1}^{N_{r}} P\left( i,j|\theta\right)j^{2}}{N_{r}(\theta)}$
   3. Gray level non-uniformity
      $gray level nonuniformity=\frac{\sum_{i=1}^{N_{g}} (\sum_{j=1}^{N_{r}} P\left( i,j|\theta\right))^{2}}{N_{r}(\theta)}$
   4. Gray level non-uniformity normalized
      $gray level nonuniformity normalized=\frac{\sum_{i=1}^{N_{g}} (\sum_{j=1}^{N_{r}} P\left( i,j|\theta\right))^{2}}{{N_{r}(\theta)}^{2}}$
   5. Run length non-uniformity
      $run length nonuniformity=\frac{\sum_{i=1}^{N_{r}} (\sum_{j=1}^{N_{g}} P\left( i,j|\theta\right))^{2}}{N_{r}(\theta)}$
   6. Run length non-uniformity normalized
      $run length nonuniformity normalized=\frac{\sum_{i=1}^{N_{r}} (\sum_{j=1}^{N_{g}} P\left( i,j|\theta\right))^{2}}{{N_{r}(\theta)}^{2}}$
   7. Run percentage
      $run percentage=\frac{N_{r}(\theta)}{N_{p}}$
   8. Gray level variance
      $gray level variance=\sum_{i=1}^{N_{g}} \sum_{j=1}^{N_{r}} p(i,j|\theta){(i-\mu)}^{2}$
      $\mu=\sum_{i=1}^{N_{g}} \sum_{j=1}^{N_{r}} p(i,j|\theta)i$
   9. Run variance
      $run variance=\sum_{i=1}^{N_{g}} \sum_{j=1}^{N_{r}} p(i,j|\theta){(j-\mu)}^{2}$
      $\mu=\sum_{i=1}^{N_{g}} \sum_{j=1}^{N_{r}} p(i,j|\theta)j$
   10. Run entropy
       $run entropy=-\sum_{i=1}^{N_{g}} \sum_{j=1}^{N_{r}} p\left( i,j|\theta\right){log}_{2}\left( p\left( i,j \right)+\epsilon\right)$
       $where \epsilon is approximate to 2.2*10e-16.$
   11. Low gray level run emphasis
       $low gray level run emphasis= \frac{\sum_{i=1}^{N_{g}} \sum_{j=1}^{N_{r}} \frac{P\left( i,j|\theta\right)}{i^{2}}}{N_{r}(\theta)}$
   12. High gray level run emphasis
       $high gray level run emphasis=\frac{\sum_{i=1}^{N_{g}} \sum_{j=1}^{N_{r}} P\left( i,j|\theta\right)i^{2}}{N_{r}(\theta)}$
   13. Short run low gray level emphasis
       $short run low gray level run emphasis= \frac{\sum_{i=1}^{N_{g}} \sum_{j=1}^{N_{r}} \frac{P\left( i,j|\theta\right)}{i^{2}j^{2}}}{N_{r}(\theta)}$
   14. Short run high gray level emphasis
       $short run high gray level run emphasis= \frac{\sum_{i=1}^{N_{g}} \sum_{j=1}^{N_{r}} \frac{P\left( i,j|\theta\right)i^{2}}{j^{2}}}{N_{r}(\theta)}$
   15. Long run low gray level emphasis
       $long run low gray level run emphasis= \frac{\sum_{i=1}^{N_{g}} \sum_{j=1}^{N_{r}} \frac{P\left( i,j|\theta\right)j^{2}}{i^{2}}}{N_{r}(\theta)}$
   16. Long run high gray level emphasis

$long run high gray level run emphasis= \frac{\sum_{i=1}^{N_{g}} \sum_{j=1}^{N_{r}} P\left( i,j|\theta\right){i^{2}j}^{2}}{N_{r}(\theta)}$

4. Neighboring gray tone difference matrix features
   1. Coarseness
      $Coarseness=\frac{1}{\sum_{i=1}^{N_{g}} p_{i}s_{i}}$
   2. Contrast
      $Contrast=(\frac{1}{N_{g,p}\left( N_{g,p-1} \right)}\sum_{i=1}^{N_{g}} \sum_{j=1}^{N_{g}} p_{i}p_{j}\left( i-j \right)^{2})(\frac{1}{N_{v,p}}\sum_{i=1}^{N_{g}} s_{i})$
      $where p_{i}{\neq0 and p}_{j}\neq0$
   3. Busyness
      $Busyness= \frac{\sum_{i=1}^{N_{g}} p_{i}s_{j}}{\sum_{i=1}^{N_{g}} \sum_{j=1}^{N_{g}} {|ip}_{i}-jp_{j}|}$
      $where p_{i}{\neq0 and p}_{j}\neq0$
   4. Complexity
      $Complexity=\frac{1}{N_{v,p}}\sum_{i=1}^{N_{g}} \sum_{j=1}^{N_{g}} |i-j|\frac{p_{i}s_{i}+p_{j}s_{j}}{p_{i}+p_{j}}$
   5. Strength

$Stength=\frac{\sum_{i=1}^{N_{g}} \sum_{j=1}^{N_{g}} {(p}_{i}+p_{j}){(i-j)}^{2}}{\sum_{i=1}^{N_{g}} s_{i}}$

$where p_{i}{\neq0 and p}_{j}\neq0$

5. Gray level dependence matrix features
   1. Small dependence emphasis
      $small dependence emphasis=\frac{\sum_{i=1}^{N_{g}} \sum_{j=1}^{N_{d}} \frac{P\left( i,j \right)}{i^{2}}}{N_{z}}$
   2. Large dependence emphasis
      $large dependence emphasis=\frac{\sum_{i=1}^{N_{g}} \sum_{j=1}^{N_{d}} P\left( i,j \right)j^{2}}{N_{z}}$
   3. Gray level non-uniformity
      $gray level nonuniformity=\frac{\sum_{i=1}^{N_{g}} (\sum_{j=1}^{N_{d}} P\left( i,j \right))^{2}}{N_{z}}$
   4. Dependence non-uniformity
      $dependence nonuniformity=\frac{\sum_{i=1}^{N_{d}} (\sum_{j=1}^{N_{g}} P\left( i,j \right))^{2}}{N_{z}}$
   5. Dependence non-uniformity normalized
      $dependence nonuniformity normalized=\frac{\sum_{i=1}^{N_{d}} (\sum_{j=1}^{N_{g}} P\left( i,j \right))^{2}}{N_{z}^{2}}$
   6. Gray level variance
      $gray level variance=\sum_{i=1}^{N_{g}} \sum_{j=1}^{N_{d}} p(i,j){(i-u)}^{2}$
      $where \mu=\sum_{i=1}^{N_{g}} \sum_{j=1}^{N_{d}} ip(i,j)$
   7. Dependence variance
      $dependence variance=\sum_{i=1}^{N_{g}} \sum_{j=1}^{N_{d}} p(i,j){(j-u)}^{2}$
      $where \mu=\sum_{i=1}^{N_{g}} \sum_{j=1}^{N_{d}} jp(i,j)$
   8. Dependence entropy
      $dependence entropy=-\sum_{i=1}^{N_{g}} \sum_{j=1}^{N_{d}} p(i,j){log}_{2}(p\left( i,j \right)+\epsilon)$
   9. Low gray level emphasis
      $low gray level emphasis= \frac{\sum_{i=1}^{N_{g}} \sum_{j=1}^{N_{d}} \frac{P\left( i,j \right)}{i^{2}}}{N_{z}}$
   10. High gray level emphasis
       $high gray level emphasis= \frac{\sum_{i=1}^{N_{g}} \sum_{j=1}^{N_{d}} P\left( i,j \right)i^{2}}{N_{z}}$
   11. Small dependence low gray level emphasis
       $small dependence low gray level emphasis=\frac{\sum_{i=1}^{N_{g}} \sum_{j=1}^{N_{d}} \frac{P\left( i,j \right)}{i^{2}j^{2}}}{N_{z}}$
   12. Small dependence high gray level emphasis
       $small dependence high gray level emphasis=\frac{\sum_{i=1}^{N_{g}} \sum_{j=1}^{N_{d}} \frac{P\left( i,j \right)i^{2}}{j^{2}}}{N_{z}}$
   13. Large dependence low gray level emphasis
       $large dependence low gray level emphasis=\frac{\sum_{i=1}^{N_{g}} \sum_{j=1}^{N_{d}} \frac{P\left( i,j \right)j^{2}}{i^{2}}}{N_{z}}$
   14. Large dependence high gray level emphasis

$large dependence high gray level emphasis=\frac{\sum_{i=1}^{N_{g}} \sum_{j=1}^{N_{d}} P\left( i,j \right)i^{2}j^{2}}{N_{z}}$

6. Wavelets-local binary pattern features

- The extraction of this kind of features is simple. The input image needs to be process by wavelet transform and local binary pattern in order. Then we extract the first-order features from these processed images.

1. Co-occurrence of Local Anisotropic Gradient Orientations (CoLlAGe) features (Gradient features)

- The method we used is the 3D version of CoLIAGe features. The algorithm is summarized below:
- Data: VOI volume $C$
- Result: 3D CoLIAGe features, $\hat{F}$ and $\tilde{F}$
- begin
- for each voxel $c\in C$, do
- Obtain gradient $\partial f_{X}\left( c \right)$, $\partial f_{Y}\left( c \right)$ and $\partial f_{Z}\left( c \right)$ along X-, Y-, and Z-axes;
- end
- for each voxel $c\in C$, do

Compute gradient vectors $\vec{\partial f_{X}}(c_{k})$, $\vec{\partial f_{Y}}(c_{k})$, and $\vec{\partial f_{Z}}\left( c_{k} \right)$ in neighborhood of $N\times N\times N$;

Obtain localized gradient vector matrix $\vec{F}=[\vec{\partial f_{X}}(c_{k})\vec{\partial f_{Y}}(c_{k})\vec{\partial f_{Z}}(c_{k})]$;

Compute dominant components $\psi\left( c_{k11} \right), \psi\left( c_{k12} \right),$ and $\psi\left( c_{k13} \right)$ by SVD of $\vec{F}$;

Obtain dominant orientations $\theta(c_{k})$ and $\phi(c_{k})$, using Eq. S1 and S2 respectively;

end

Compute co-occurrence matrix $\hat{M}$ from $\theta(c_{k})$ and $\tilde{M}$ from $\phi(c_{k})$ using Eq. S3 and S4;

Compute the $\hat{\varepsilon}$ and $\tilde{\varepsilon}$ from $\hat{M}$ and $\tilde{M}$ based on Haralick features, respectively;

Compute the histogram features $\hat{F}$ and $\tilde{F}$ from $\hat{\varepsilon}$ and $\tilde{\varepsilon}$, respectively;

end

Eq. 1

Eq. 2

Eq. 3

Eq. 4
